# Hemoglobin Uptake by *Paracoccidioides* spp. Is Receptor-Mediated

**DOI:** 10.1371/journal.pntd.0002856

**Published:** 2014-05-15

**Authors:** Elisa Flávia Luiz Cardoso Bailão, Juliana Alves Parente, Laurine Lacerda Pigosso, Kelly Pacheco de Castro, Fernanda Lopes Fonseca, Mirelle Garcia Silva-Bailão, Sônia Nair Báo, Alexandre Melo Bailão, Marcio L. Rodrigues, Orville Hernandez, Juan G. McEwen, Célia Maria de Almeida Soares

**Affiliations:** 1 Laboratório de Biologia Molecular, Instituto de Ciências Biológicas, Universidade Federal de Goiás, Goiânia, Goiás, Brazil; 2 Unidade Universitária de Iporá, Universidade Estadual de Goiás, Iporá, Goiás, Brazil; 3 Programa de Pós Graduação em Patologia Molecular, Faculdade de Medicina, Universidade de Brasília, Brasília, Distrito Federal, Brazil; 4 Instituto de Microbiologia Professor Paulo de Góes, Universidade Federal do Rio de Janeiro, Brazil; 5 Laboratório de Microscopia Eletrônica, Universidade de Brasília, Distrito Federal, Brazil; 6 Fundação Oswaldo Cruz – Fiocruz, Centro de Desenvolvimento Tecnológico em Saúde (CDTS), Rio de Janeiro, Brazil; 7 Unidad de Biología Celular y Molecular, Corporación para Investigaciones Biológicas (CIB), Medellín, Colombia; 8 Facultad de Ciencias de la Salud, Institución Universitaria Colegio Mayor de Antioquia, Medellín, Colombia; 9 Facultad de Medicina, Universidad de Antioquia, Medellín, Colombia; University of California San Diego School of Medicine, United States of America

## Abstract

Iron is essential for the proliferation of fungal pathogens during infection. The availability of iron is limited due to its association with host proteins. Fungal pathogens have evolved different mechanisms to acquire iron from host; however, little is known regarding how *Paracoccidioides* species incorporate and metabolize this ion. In this work, host iron sources that are used by *Paracoccidioides* spp. were investigated. Robust fungal growth in the presence of the iron-containing molecules hemin and hemoglobin was observed. *Paracoccidioides* spp. present hemolytic activity and have the ability to internalize a protoporphyrin ring. Using real-time PCR and nanoUPLC-MS^E^ proteomic approaches, fungal growth in the presence of hemoglobin was shown to result in the positive regulation of transcripts that encode putative hemoglobin receptors, in addition to the induction of proteins that are required for amino acid metabolism and vacuolar protein degradation. In fact, one hemoglobin receptor ortholog, Rbt5, was identified as a surface GPI-anchored protein that recognized hemin, protoporphyrin and hemoglobin *in vitro*. Antisense RNA technology and *Agrobacterium tumefaciens*-mediated transformation were used to generate mitotically stable *Pbrbt5* mutants. The knockdown strain had a lower survival inside macrophages and in mouse spleen when compared with the parental strain, which suggested that Rbt5 could act as a virulence factor. In summary, our data indicate that *Paracoccidioides* spp. can use hemoglobin as an iron source most likely through receptor-mediated pathways that might be relevant for pathogenic mechanisms.

## Author Summary

Fungal infections contribute substantially to human morbidity and mortality. During infectious processes, fungi have evolved mechanisms to obtain iron from high-affinity iron-binding proteins. In the current study, we demonstrated that hemoglobin is the preferential host iron source for the thermodimorphic fungus *Paracoccidioides* spp. To acquire hemoglobin, the fungus presents hemolytic activity and the ability to internalize protoporphyrin rings. A putative hemoglobin receptor, Rbt5, was demonstrated to be GPI-anchored at the yeast cell surface. Rbt5 was able to bind to hemin, protoporphyrin and hemoglobin *in vitro*. When *rbt5* expression was inhibited, the survival of *Paracoccidioides* sp. inside macrophages and the fungal burden in mouse spleen diminished, which indicated that Rbt5 could participate in the establishment of the fungus inside the host. Drugs or vaccines could be developed against *Paracoccidioides* spp. Rbt5 to disturb iron uptake of this micronutrient and, thus, the proliferation of the fungus. Moreover, this protein could be used in routes to introduce antifungal agents into fungal cells.

## Introduction

Iron is an essential micronutrient for almost all organisms, including fungi. Because iron is a transition element, iron can participate as a cofactor in a series of biological processes, such as respiration and amino acid metabolism, as well as DNA and sterol biosynthesis [1]. However, at high levels, iron can be toxic, generating reactive oxygen species (ROS). The regulation of iron acquisition in fungi is one of the most critical steps in maintaining iron homeostasis because these micro-organisms have not been described as possessing a regulated mechanism of iron egress [2].

The mammal host actively regulates intracellular and systemic iron levels as a mechanism to contain microbial infection and persistence. Because of this, microbial iron acquisition is an important virulence attribute. One strategy to protect the body against iron-dependent ROS cascades and to keep iron away from microorganisms is to tightly bind the metal to many proteins, including hemoglobin, ferritin, transferrin and lactoferrin [3]. In human blood, 66% of the total circulating body iron is bound to hemoglobin. Each hemoglobin molecule possesses four heme groups, and each heme group contains one ferrous ion (Fe^2+^) [4]. Iron that is bound to the glycoprotein transferrin, which presents two ferric ion (Fe^3+^) high affinity binding sites, circulates in mammalian plasma [5]. Lactoferrin is present in body fluids, such as serum, milk, saliva and tears [6]. Additionally, similar to transferrin, lactoferrin possesses two Fe^3+^ binding sites [7]. Lactoferrin functions as a defense molecule due to its ability to sequester iron [8]. Although these proteins are important in sequestering extracellular iron, ferritin is primarily an intracellular iron storage protein [9] and is composed of 24 subunits that are composed of approximately 4500 Fe^3+^ ions [10].

Most microorganisms can acquire iron from the host by utilizing high-affinity iron-binding proteins. Preferences for specific host iron sources and strategies to gain iron that is linked to host proteins are under study. It has been revealed, for example, that *Staphylococcus aureus* preferentially uses iron from heme rather than from transferrin during early infection [11]. However, thus far, there is a scarcity of data from pathogenic fungi. It has been suggested that *Cryptococcus neoformans* preferentially uses transferrin as the host iron source through a reductive iron uptake system because Cft1 (*Cryptococcus* Fe Transporter) is required for transferrin utilization and is essential for full virulence [12]. *Histoplasma capsulatum* seems to preferentially use transferrin as the host iron source but also uses hemin and ferritin [13], [14]. *Candida albicans* can also mediate iron acquisition from transferrin [15]. Moreover, the Als3 (Agglutinin-like sequence) protein functions as a receptor at the surface of *C. albicans* hyphae, which could support iron acquisition from ferritin [16].

The strategy for iron acquisition from hemoglobin by *C. albicans* is the best characterized. *C. albicans* presents hemolytic activity and utilizes hemin and hemoglobin as iron sources [17]–[20]. For erythrocyte lyses, *C. albicans* most likely possesses a hemolytic factor that is attached to the fungal cell surface [21]. After hemoglobin release, surface receptors, e.g., Rbt5 (Repressed by Tup1), Rbt51, Wap1/Csa1 (*Candida* Surface Antigen), Csa2 and Pga7 (Predicted GPI-Anchored), could function in the uptake of hemoglobin [19]. Those receptors possess a CFEM domain, which is characterized by a sequence of eight spaced cysteine residues [22], that might bind heme through the iron atom [23]. It has been demonstrated that *rbt5* and *wap1* are transcriptionally activated during low iron conditions (10 µM) in comparison with high iron conditions (100 µM), which indicates that these encoding proteins are important in high-affinity iron uptake pathways [24]. Rbt5, which is a glycosylphosphatidylinositol (GPI)-anchored protein, appears to have a central role in hemin/hemoglobin uptake because the *rbt5* deletion impaired *C. albicans* growth in the presence of hemin and hemoglobin as iron sources [19]. However, *rbt5* deletion did not affect *C. albicans* virulence in a mouse model of systemic infection or during rabbit corneal infection [25], which indicates that other compensatory mechanisms could act in the absence of Rbt5 [19]. It is suggested that after hemoglobin binds to Rbt5, the host iron source is internalized by endocytosis into vacuoles [20].

It has been proposed that the *C. neoformans* mannoprotein cytokine-inducing glycoprotein (Cig1) acts as a hemophore at the cell surface, which sequesters heme for internalization via a receptor that has not yet been described [26]. After heme binding, the molecule is most likely internalized via endocytosis with the participation of the ESCRT pathway [27], as described for *C. albicans* [20]. In *C. neoformans*, the deletion of *vps23*, which is an ESCRT-I component, resulted in a growth defect on heme and reduced susceptibility to non-iron metalloporphyrins, which have heme-uptake dependent toxicity, indicating that the endocytosis pathway is important for hemoglobin utilization by this fungus [27].

In the host, macrophages play an important role in maintaining adequate levels of plasma iron. Those cells phagocyte aged or damaged erythrocytes and internally recycle iron from senescent erythrocytes [5]. Macrophages are the first host defense cells that interact with *Paracoccidioides* spp. [28], which is a complex of two suggested species (*P. brasiliensis* and *P. lutzii*) of thermodimorphic fungi [29]. Here, this complex is designated as *Paracoccidioides*. All strains of *Paracoccidioides* that have been described thus far are causative agents of paracoccidioidomycosis (PCM) [29], which is a systemic mycosis [30]. Non-activated macrophages are permissive to intracellular *Paracoccidioides* multiplication, functioning as a protected environment against complement systems, antibodies and innate immune components and thus leading to fungal dissemination from the lungs to other tissues [31], [32]. Possible strategies that are used by *Paracoccidioides* to survive inside macrophages include (i) the downregulation of macrophage genes that are involved in the inflammatory response and in the activation against pathogens [33], [34], (ii) the inhibition of phagosome-endosome fusion [35] and (iii) the detoxification of ROS that are produced by the phagocyte NADPH oxidase system [36]. Moreover, iron availability inside monocytes is required for *Paracoccidioides* survival because the effect of chloroquine on fungal survival is reversed by FeNTA, which is an iron compound that is soluble in the neutral to alkaline pH range [37].

The host iron sources that are used by *Paracoccidioides* have not been established to date. In this work, we demonstrate that *Paracoccidioides* can use hemoglobin as an iron source through a receptor-mediated pathway during infection. This observation unravels new mechanisms by which *Paracoccidioides* species might interfere with the physiology of host tissues.

## Materials and Methods

### Ethics statement

All animals were treated in accordance with the guidelines provided by the Ethics Committee on Animal Use from Universidade Federal de Goiás based on the International Guiding Principles for Biomedical Research Involving Animals (http://www.cioms.ch/images/stories/CIOMS/guidelines/1985_texts_of_guidelines.htm) and their use was approved by this committee (131/2008).

### Strains and growth conditions

*Paracoccidioides* strains *Pb*01 (ATCC MYA-826; *Paracoccidioides lutzii*) [29], *Pb*18 (ATCC 32069; *Paracoccidioides brasiliensis*, phylogenetic species S1) and *Pb*339 (ATCC 200273; *Paracoccidioides brasiliensis*, phylogenetic species S1) [38] were used in this work. The fungus was maintained in brain heart infusion (BHI) medium, which was supplemented with 4% (w/v) glucose at 36°C to cultivate the yeast form. For growth assays, *Paracoccidioides* yeast cells were incubated in chemically defined MMcM medium [39] with no iron addition for 36 h at 36°C under rotation to deplete intracellular iron storage. Cells were collected and washed twice with phosphate buffered saline solution 1X (1X PBS; 1.4 mM KH_2_PO_4_, 8 mM Na_2_HPO_4_, 140 mM NaCl, 2.7 mM KCl; pH 7.3). Cell suspensions were serially diluted and spotted on plates with MMcM medium, which contained 50 µM of bathophenanthroline disulfonic acid (BPS) that was supplemented or not (no iron condition) with different iron sources: 30 µM inorganic iron [Fe(NH_4_)_2_(SO_4_)_2_], 30 µM hemoglobin, 120 µM hemin, 30 µg/ml ferritin, 30 µM transferrin or 3 µM lactoferrin. All host iron sources were purchased from Sigma-Aldrich, St. Louis, MO, USA.

### Fluorescence microscopy

*Paracoccidioides* yeast cells were maintained in MMcM medium for 36 h. Those cells were pre-incubated or not with hemoglobin for 1 h at room temperature. After this time, the cells were incubated on MMcM medium, which was supplemented or not with different concentrations of zinc protoporphyrin IX (zinc-PPIX) (Sigma-Aldrich, St. Louis, MO, USA) for different times at 36°C under rotation. Cells were collected, washed twice with 1X PBS and observed by live fluorescence microscopy using an Axio Scope A1 microscope with a 40x objective and the software AxioVision (Carl Zeiss AG, Germany). The Zeiss filter set 15 was used to detect intrinsic zinc-PPIX fluorescence. The camera exposition time was fixed in 710 ms for all pictures. The fluorescence background was determined in the absence of zinc-PPIX in the MMcM medium.

### Hemolytic activity of *Paracoccidioides*

The hemolytic activity of *Paracoccidioides* was evaluated as described previously [17], with modifications. Briefly, the fungus was cultivated in MMcM medium with no iron addition for 36 h at 36°C, under rotation. After this period, the yeast cells were harvested and washed twice with 1X PBS. Then, 10^7^ cells were incubated with 10^8^ sheep erythrocytes (Newprov Ltda, Pinhais, Paraná, Brazil) for 2 h, at 36°C in 5% CO_2_. As negative or positive controls, respectively, erythrocytes were incubated with 1X PBS or water. After incubation, the cells were resuspended by gentle pipetting, and then pelleted by brief centrifugation. The optical densities of the supernatants were determined using an ELISA plate reader at 405 nm. The experiment was performed in triplicate, and the average of the optical density was obtained for each condition. The average optical density of each condition was used to calculate the relative hemolysis of the experimental conditions or the negative control against the positive control. The relative hemolysis data were plotted in a bar graph. Student's t-test was applied to compare the experimental values to the negative control values.

### *In silico* analysis of *Paracoccidioides* putative hemoglobin receptors

The amino acid sequences of putative members of the *Paracoccidioides* hemoglobin receptor family were obtained from the Dimorphic Fungal Database of the Broad Institute site at (http://www.broadinstitute.org/annotation/genome/paracoccidioides_brasiliensis/MultiHome.html) based on a homology search. The sequences for *Pb*01 Rbt5, Wap1 and Csa2 have been submitted to GenBank with the following respective accession numbers: XP_002793022, XP_002795519 and XP_002797192. For *Pb*18 Wap1, the accession number is EEH49284. And for *Pb*03 Rbt51 and Csa2, the accession numbers are, respectively, EEH22388 and EEH19315. SMART (http://smart.embl-heidelberg.de/), SignalP 4.1 Server (http://www.cbs.dtu.dk/services/SignalP/) and big-PI Fungal Predictor (http://mendel.imp.ac.at/gpi/fungi_server.html) protein analysis tools were used to search for conserved domains, signal peptides and GPI modification sites, respectively, in *Paracoccidioides* and *C. albicans* sequences. The amino acid sequences of *Paracoccidioides* and *C. albicans* orthologs were aligned using the CLUSTALX2 program [40].

### RNA extraction and quantitative real time PCR (qRT-PCR)

*Pb*01 yeast cells were incubated in MMcM medium without iron or in MMcM medium supplemented with different iron sources: 10 or 100 µM inorganic iron or 10 µM hemoglobin. Cells were harvested after 30, 60 or 120 min of incubation, and total RNA was extracted using TRIzol (TRI Reagent, Sigma-Aldrich, St. Louis, MO, USA) and mechanical cell rupture (Mini-Beadbeater - Biospec Products Inc., Bartlesville, OK). After *in vitro* reverse transcription (SuperScript III First-Strand Synthesis SuperMix; Invitrogen, Life Technologies), the cDNAs were submitted to a qRT-PCR reaction, which was performed using SYBR Green PCR Master Mix (Applied Biosystems, Foster City, CA) in a StepOnePlus Real-Time PCR System (Applied Biosystems Inc.). The expression values were calculated using the transcript that encoded *alpha tubulin* (XM_002796593) as the endogenous control as previously reported [41]. The primer pairs for qRT-PCR were designed such that one primer in each pair spanned an intron, which prevented genomic DNA amplification. The sequences of the oligonucleotide primers that were used were as follows: *rbt5*-S, 5′- ATATCCCACCTTGCGCTTTGA -3′; *rbt5*-AS, 5′- GGGCAGCAACGTCGCAAGA -3′; *wap1*-S, 5′- AAGTCTGTGATAGTGCTGGAG - 3′; *wap1*-AS, 5′- AGGGGGTTCAGGGAGAGGA -3′; *csa2*-S, 5′- GCAAAATTAAAGAATCTCTCACG -3′; *csa2*-AS, 5′- ATGAAACGGCAAATCCCACCA-3′; *alpha-tubulin*-S, 5′- ACAGTGCTTGGGAACTATACC -3′; *alpha-tubulin*-AS, 5′- GGGACATATTTGCCACTGCC -3′. The annealing temperature for all primers was 62°C. The qRT-PCR reaction was performed in triplicate for each cDNA sample, and a melting curve analysis was performed to confirm single PCR products. The relative standard curve was generated using a pool of cDNAs from all the conditions that were used, which was serially diluted 1∶5 to 1∶625. Relative expression levels of transcripts of interest were calculated using the standard curve method for relative quantification [42]. Student's t-test was applied in the statistical analyses.

### Sample preparation, nanoUPLC-MS^E^ acquisition and protein classification

*Pb*01 yeast cells were cultivated in MMcM medium with 10 µM inorganic iron [Fe(NH_4_)_2_(SO_4_)_2_] or with 10 µM bovine hemoglobin (H2500-Sigma-Aldrich, St. Louis, MO, USA) at 36°C under constant agitation. After 48 h, the cells were harvested, and the cell rupture was performed as described above, in the presence of Tris-Ca buffer (Tris-HCl 20 mM, pH 8.8; CaCl2 2 mM) with 1% proteases inhibitor (Protease Inhibitor mix 100x, Amersham). The mixtures were centrifuged at 12,000 g at 4°C for 10 min. The supernatant was collected and centrifuged again, at the same conditions for 20 min. Then, the protein extracts were washed twice with 50 mM NH_4_HCO_3_ buffer and concentrated using a 10 kDa molecular weight cut-off in an Ultracel regenerated membrane (Amicon Ultra centrifugal filter, Millipore, Bedford, MA, USA). The proteins extracts concentration were determined using the Bradford assay [43]. These extracts were prepared as previously described [44] for analyses using nano-scale ultra-performance liquid chromatography combined with mass spectrometry with data-independent acquisitions (nanoUPLC-MS^E^). In this way, the trypsin-digested peptides were separated using a nanoACQUITY UPLC System (Waters Corporation, Manchester, UK). The MS data that were obtained via nanoUPLC-MS^E^ were processed and examined using the ProteinLynx Global Server (PLGS) version 2.5 (Waters Corporation, Manchester, UK). Protein identification and quantification level analyses were performed as described previously [45]. The observed intensity measurements were normalized with the identified peptides of the digested internal standard rabbit phosphorylase. For protein identification, the *Paracoccidioides* genome database was used. Protein tables that were generated by PLGS were merged using the FBAT software [46], and the dynamic range of the experiment was calculated using the MassPivot software (kindly provided by Dr. Andre M. Murad) by setting the minimum repeat rate for each protein in all replicates to 2 as described previously [45]. Proteins were considered regulated when p<0.05 (determined by PLGS) and when the fold change between protein quantification in the presence of hemoglobin x presence of inorganic iron was ±0.2. Proteins were classified according to MIPS functional categorization (http://mips.helmholtz-muenchen.de/proj/funcatDB/) with the help of the online tools UniProt (http://www.uniprot.org/), PEDANT (http://pedant.helmholtz-muenchen.de/pedant3htmlview/pedant3view?Method=analysis&Db=p3_r48325_Par_brasi_Pb01) and KEGG (http://www.genome.jp/kegg/). Graphics that indicated the quality of the proteomic data were generated using the Spotfire software (http://spotfire.tibco.com/).

### Expression and purification of recombinant Rbt5

Oligonucleotide primers were designed to amplify the 585 bp complete coding region of Rbt5: *rbt5*-S, 5′- GGTGTCGACCAGCTCCCTAATATCCCAC -3′; *rbt5*-AS, 5′- GGTGCGGCCGCGACATAATTTACAGGTAAGC -3′ (underlined regions correspond to *Not*I and *Sal*I restriction sites, respectively). The PCR product was subcloned into the *Not*I/*Sal*I sites of pGEX-4T-3 (GE Healthcare). The DNA was sequenced on both strands and was used to transform the *E. coli* C41 (DE3). The transformed cells were grown at 37°C, and protein expression was induced by the addition of 1 mM isopropyl β-D- thiogalactopyranoside (IPTG) for 5 h. The bacterial extract was centrifuged at 2,700 g and was resuspended in 1X PBS. The fusion protein Rbt5 was expressed in the soluble form in the heterologous system and was purified by affinity chromatography under non-denaturing conditions using glutathionesepharose 4B resin (GE Healthcare). Subsequently, the fusion protein was cleaved by the addition of thrombin protease (50 U/ml). The purity and size of the recombinant protein were evaluated by resuspending the protein in SDS-loading buffer [50 mM Tris-HCl, pH 6.8; 100 mM dithiothreitol, 2% (w/v) SDS; 0.1% (w/v) bromophenol blue; 10% (v/v) glycerol]. Subsequently the sample was boiled for 5 min, followed by running the purified molecule on a 12% sodium dodecyl sulfate-polyacrylamide gel electrophoresis (SDS-PAGE) and finally, staining with Coomassie blue.

### Antibody production

The purified Rbt5 was used to generate a specific rabbit polyclonal serum. Rabbit preimmune serum was obtained and stored at −20°C. The purified recombinant protein (300 µg) was injected into rabbit with Freund's adjuvant three times at 10-day intervals. The obtained serum was sampled and stored at −20°C.

### Cell wall protein extractions and enzymatic treatments

Yeast cells were frozen in liquid nitrogen and disrupted by using a mortar and pestle. This procedure was performed until the cells completely ruptured, which was verified by optical microscopic analysis. The ground material was lyophilized, weighed, and resuspended in 25 µl Tris buffer (50 mM Tris-HCl, pH 7.8) for each milligram of dry weight as described previously [47]. The supernatant was separated from the cell wall fraction by centrifugation at 10,000 g for 10 min at 4°C. To remove proteins that were not covalently linked and intracellular contaminants, the isolated cell wall fraction was washed extensively with 1 M NaCl, was boiled three times in SDS-extraction buffer (50 mM Tris-HCl, pH 7.8, 2% [w/v] SDS, 100 mM Na-EDTA, 40 mM β-mercaptoethanol) and pelleted by centrifugation at 10,000 g for 10 min [48]. The washed pellet containing the cell wall enriched fraction was washed six times with water, lyophilized, and weighed. The cell wall fraction, which was prepared as described above, was treated with hydrofluoric acid-pyridine (HF-pyridine) (10 µl for each milligram of dry weight of cell walls) for 4 h at 0°C [49], [50]. After centrifugation, the supernatant that contained the HF-pyridine extracted proteins was collected, and HF-pyridine was removed by precipitating the supernatant in 9 volumes of methanol buffer (50% v/v methanol, 50 mM Tris-HCl, pH 7.8) at 0°C for 2 h. The pellet was washed three times in methanol buffer and resuspended in approximately 10 times the pellet volume in SDS-loading buffer, as described previously [50].

### Western blotting analysis

Twenty micrograms of protein samples were loaded onto a 12% SDS-PAGE gel and were separated by electrophoresis. Proteins were transferred from gels to nitrocellulose membrane at 20 V for 16 h in buffer that contained 25 mM Tris-HCl pH 8.8, 190 mM glycine and 20% (v/v) methanol. Membranes were stained with Ponceau red to confirm complete protein transfer. Next, each membrane was submerged in blocking buffer [1X PBS, 5% (w/v) non-fat dried milk, 0.1% (v/v) Tween-20] for 2 h. Membranes were washed with wash buffer [1X PBS, 0.1% (v/v) Tween-20] and incubated with primary antibody, which was used at a 1/3,000 (v/v) ratio of antibody to buffer, for 1 h at room temperature. This step was followed by three 15 min washes with wash buffer. Membranes were incubated with the conjugated secondary antibody [anti-rabbit immunoglobulin G coupled to alkaline phosphatase (Sigma-Aldrich, St. Louis, MO, USA)] in a 1/5,000 (v/v) ratio, for 1 h at room temperature, and developed with 5-bromo-4-chloro-3-indolylphosphate–nitroblue tetrazolium (BCIP-NBT). Reactions were also performed with sera from patients with PCM, sera from control individuals (all diluted 1∶100) and with 1 µg of purified recombinant Rbt5. After incubation with peroxidase conjugate anti-human IgG (diluted 1∶1000), the reaction was developed with hydrogen peroxide and diaminobenzidine (Sigma-Aldrich, St. Louis, MO, USA) as the chromogenic reagent.

### Transmission electron microscopy of *Paracoccidioides* yeast cells and immunocytochemistry of Rbt5

For the ultrastructural and immunocytochemistry studies, the protocols that were previously described by Lima and colleagues [51] were employed. Transmission electron microscopy was performed using thin sections from *Pb*01 yeast that were fixed in 2% (v/v) glutaraldehyde, 2% (w/v) paraformaldehyde and 3% (w/v) sucrose in 0.1 M sodium cacodylate buffer pH 7.2. The samples were post-fixed in a solution that contained 1% (w/v) osmium tetroxide, 0.8% (w/v) potassium ferricyanide and 5 mM CaCl_2_ in sodium cacodylate buffer, pH 7.2. The material was embedded in Spurr resin (Electron Microscopy Sciences, Washington, PA). Ultrathin sections were stained with 3% (w/v) uranyl acetate and lead citrate. For immunolabeling, the cells were fixed in a mixture that contained 4% (w/v) paraformaldehyde, 0.5% (v/v) glutaraldehyde and 0.2% (w/v) picric acid in 0.1 M sodium cacodylate buffer at pH 7.2 for 24 h at 4°C. Free aldehyde groups were quenched with 50 mM ammonium chloride for 1 h. Block staining was performed in a solution containing 2% (w/v) uranyl acetate in 15% (v/v) acetone. After dehydration, samples were embedded in LR Gold resin (Electron Microscopy Sciences, Washington, PA.). For ultrastructural immunocytochemistry studies, the ultrathin sections were incubated for 1 h with the polyclonal antibody raised against the recombinant *Pb*01 Rbt5, which was diluted 1∶100, and for 1 h at room temperature with the labeled secondary antibody anti-rabbit IgG Au-conjugated (10 nm average particle size; 1∶20 dilution; Electron Microscopy Sciences, Washington, PA). The nickel grids were stained as described above and observed using a Jeol 1011 transmission electron microscope (Jeol, Tokyo, Japan). Controls were incubated with a rabbit preimmune serum, which was diluted 1∶100, followed by incubation with the labeled secondary antibody.

### Hemin-agarose binding assay

The recombinant proteins Rbt5 was pre-incubated with hemoglobin or with 1X PBS, as a control, for 1 h at room temperature. After this time, the protein was incubated with a hemin-agarose resin (Sigma-Aldrich, St. Louis, MO, USA) for 1 h at 4°C. The recombinant protein, Enolase, previously obtained in our laboratory [52], was independently incubated with the hemin-agarose resin to function as a specificity control. After the batch binding strategy, the resin was washed three times with cold 1X PBS, resuspended in SDS-loading buffer and boiled for 5 min to elute proteins that were bound to the resin. The samples were submitted to SDS-PAGE, and the proteins were transferred to nitrocellulose membranes as cited above. For Western blot analyses, the primary antibodies anti-Rbt5 and anti-Enolase were used at 1/3,000 and 1/40,000 (v/v) ratios, respectively, and developed with BCIP-NBT, as cited above.

### Flow cytometry

Rbt5 binding affinities for hemoglobin and protoporphyrin were evaluated by flow cytometry. Yeast cells of *Paracoccidioides* [strains *Pb*01, *Pb*339 (*Pb*Wt), *Pb*rbt5-aRNA and *Pb*Wt+EV] were cultivated as described above, washed with 1X PBS and blocked for 1 h at room temperature in 1X PBS, which was supplemented with 1% bovine serum albumin (PBS-BSA). Fungal cells were then separated in two groups: the first group was initially treated with 20 µM protoporphyrin or 10 µM hemoglobin and further incubated with the anti-Rbt5 antibodies and an Alexa Fluor 488-labeled anti-rabbit IgG (10 µg/ml). The second group was sequentially incubated with primary and secondary antibodies as described above. The cells were then treated with 20 µM protoporphyrin or with 10 µM hemoglobin. All incubations were performed for 30 min at 37°C, followed by washing with 1X PBS. Control cells were not exposed to hemoglobin or to protoporphyrin. Fluorescence levels of yeast cells were analyzed using a FACSCalibur (BD Biosciences) flow cytometer, and the data were processed using the FACS Express software.

### Construction of *P. brasiliensis* Rbt5 antisense-RNA strain

The antisense-RNA (aRNA) strategy was used as described previously [53], [54]. Briefly, DNA from wild-type *Pb*339 (*Pb*Wt) exponentially growing yeast cells was obtained after cell rupture as described above. Platinum Taq DNA Polymerase High Fidelity (Invitrogen, USA) and the oligonucleotides as*rbt5*-S, 5′ - CCGCTCGAGCGGTCTCGGAAACGACGGGTGC - 3′ and as*rbt5*-AS, 5′ - GGCGCGCCCGCAAGATTTCTCAACGCAAG - 3′ were employed to amplify aRNA from *Pb*Wt *rbt5* DNA. Plasmid construction for aRNA gene repression and *A. tumefaciens*-mediated transformation (ATMT) of *Pb*Wt was performed as previously described [55], [56]. The amplified *rbt5*-aRNA fragments were inserted into the pCR35 plasmid, which was flanked by the calcium-binding protein promoter region (P-*cbp-1*) from *H. capsulatum* and by the cat-B termination region (T-*cat-B*) of *Aspergillus fumigatus* [57]. The pUR5750 plasmid was used as a parental binary vector to harbor the aRNA cassette within the transfer DNA (T-DNA). The constructed binary vectors were introduced into *A. tumefaciens* LBA1100 ultracompetent cells by electroporation [58] and were isolated by kanamycin selection (100 mg/ml). The *A. tumefaciens* cells that were positive for pUR5750 transformation were used to perform the ATMT of *Paracoccidioides* yeast cells. The hygromycin (Hyg)-resistance gene, *hph*, from *E. coli* was used as a selection mark and was flanked by the glyceraldehyde-3-phosphate dehydrogenase promoter region (P-*gapdh*) and the trpC termination region (T-*trpC*) from *Aspergillus nidulans*. The selection of transformants (*Pbrbt5*-aRNA) was performed in BHI solid media with Hyg B (75 µg/ml Hyg) during 15 days of incubation at 36°C. Randomly selected Hyg resistant transformants were tested for mitotic stability by subculturing the fungus three times in Hyg 75 µg/ml and three more times in Hyg 150 µg/ml. *Paracoccidioides* yeast cells were also transformed with the empty parental vector pUR5750 (*Pb*Wt+EV) as a control during the assays that were performed in this study. The investigation of *rbt5* gene expression was performed by qRT-PCR after consecutive subculturing.

### Macrophage infection

Macrophages from the cell line J774 A.1 (BCRJ Cell Bank, Rio de Janeiro, accession number 0121), which were maintained in RPMI medium (RPMI 1640, Vitrocell, Brazil) that was supplemented with 10% (v/v) fetal bovine serum (FBS) at 37°C in 5% CO_2_, were used in this assay. In total, 1×10^6^ macrophages were seeded into each well of a 24-well tissue culture plate, and 100 U/ml of murine IFN-γ (PeproTech, Rocky Hill, New Jersey, USA) was added for 24 h at 37°C in 5% CO_2_ for macrophage activation as described previously [59]. Prior to co-cultivation, *Paracoccidioides* yeast cells (*Pb*Wt, *Pbrbt5*-aRNA and *Pb*Wt+EV) were cultivated in BHI liquid medium for 72 h at 36°C. For infection, 2.5×10^6^ *Paracoccidioides* yeast cells for each strain were added to the macrophages independently. The cells were co-cultivated for 24 h at 37°C in 5% CO_2_ to allow fungal internalization. Infected macrophages were first washed three times with 1X PBS, and then macrophages were lysed with distilled water. Dilutions of the lysates were plated in BHI medium, which was supplemented with 5% (v/v) fetal bovine serum (FBS), at 36°C. Colony forming units (CFUs) were counted after growth for 10 days. CFUs were expressed as the mean value ± the standard error of the mean (SEM) from triplicates, and statistical analyses were performed using Student's t-test.

### BALB/c mice infection

For the mouse infection experiment, *Pb*Wt, *Pbrbt5*-aRNA and *Pb*Wt+EV were cultivated for 48 h in BHI medium, which was supplemented with 4% glucose. Thirty-day-old male BALB/c mice (n = 4) were inoculated intraperitoneally with 10^7^ yeast cells of each strain independently, as previously described [60]. After 2 weeks of infection, mouse spleens were removed and were homogenized using a grinder in 3 mL of sterile 0.9% (w/v) NaCl. In total, 50 µl of the homogenized sample was plated on BHI agar, which was supplemented with 4% (v/v) fetal calf serum and 4% (w/v) glucose. The plates were prepared in triplicates for each organ of each animal and were incubated at 36°C. After 15 days, the CFUs for each organ that was infected with each strain were determined by counting, and a mean for each condition was obtained. The data were expressed as the mean value ± the SEM from quadruplicates, and statistical analyses were performed using Student's t-test.

## Results

### Hemoglobin and heme group uptake by *Paracoccidioides*

**Figure 1:**
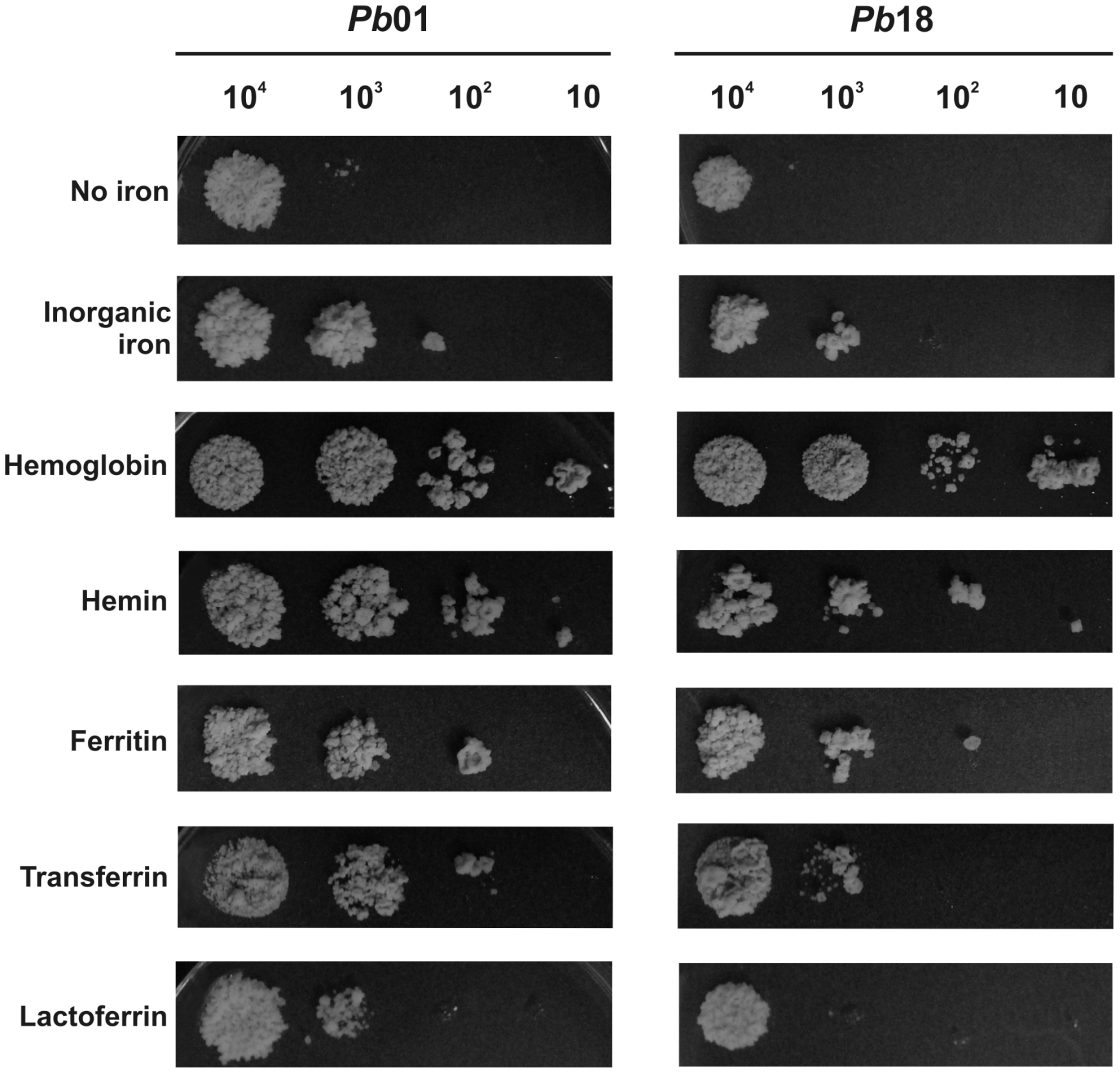
Effect of different iron sources on the growth of *Paracoccidioides* yeast cells. *Pb*01 and *Pb*18 cell cultures were collected after 36 h of iron scarcity, washed and ten-fold serial dilutions of cell suspensions (10^4^ to 10 cells) were spotted on MMcM medium plates, which were supplemented with 50 µM BPS, an iron chelator. As indicated, different iron sources were added or not (no iron condition): 30 µM inorganic iron, 30 µM hemoglobin, 120 µM hemin, 30 µg/µl ferritin, 30 µM transferrin or 3 µM lactoferrin.

The *Paracoccidioides* strains *Pb*01 and *Pb*18 were grown in the absence of iron (by adding 50 µM BPS, an iron chelator), or in the presence of different iron sources, after 36 h of iron scarcity to deplete intracellular iron storage (**Figure 1**). The host iron sources that were tested in this work included hemoglobin, ferritin, transferrin and lactoferrin. An inorganic iron source, ferrous ammonium sulfate, was also used. In all conditions, 50 µM BPS was added to verify that the chelator itself does not interfere with *Paracoccidioides* growth. Although some subtle differences were observed in the growth profiles, *Pb*01 and *Pb*18 were able to grow efficiently in the presence of different host iron sources, primarily hemoglobin and ferritin for both strains, and transferrin primarily for the *Pb*01 strain. In iron-depleted medium, *Paracoccidioides* grew poorly. Notably, both *Pb*01 and *Pb*18 presented a robust growth in the presence of hemoglobin or hemin as sole iron sources (**Figure 1**), which suggested that the increased growth in the presence of hemoglobin was not only due to the amino acid portion but also due to the heme group. These results indicate that hemoglobin could represent an important iron source for *Paracoccidioides* in the host environment.

**Figure 2:**
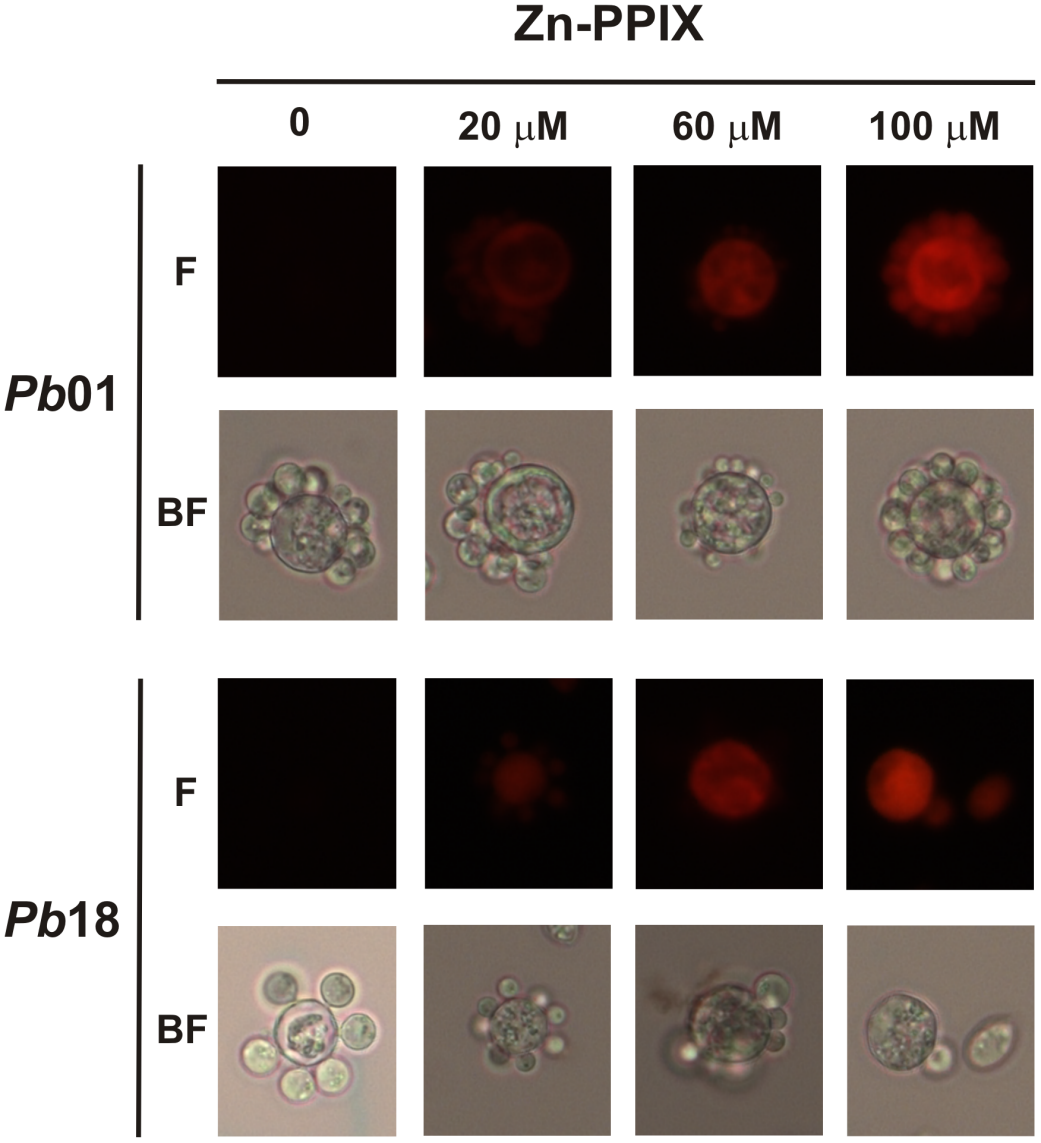
*Paracoccidioides* can internalize protoporphyrin rings. Iron deprived *Pb*01 and *Pb*18 yeast cells were incubated in MMcM medium supplemented or not (0) with different zinc protoporphyrin IX (Zn-PPIX) concentrations (20–100 µM) for 2 h. After this period, the cells were washed twice, and observed by bright field microscopy (BF) and by live fluorescence microscopy (F).

**Figure 3:**
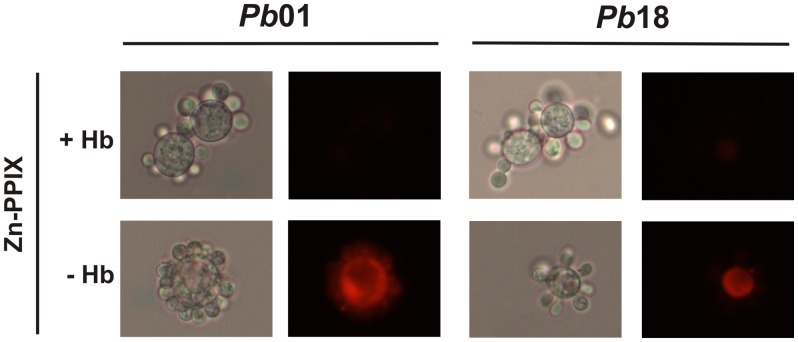
Hemoglobin can block the Zn-PPIX internalization by *Paracoccidioides*. Iron deprived *Pb*01 and *Pb*18 yeast cells were pre-incubated (+) or not (−) with hemoglobin (Hb) for 1 h. After, the cells were incubated in MMcM medium supplemented with 60 µM zinc protoporphyrin IX (Zn-PPIX) for 2 h. After this period, the cells were washed twice and observed by bright field microscopy (left panels for each strain) and by live fluorescence microscopy (right panels for each strain).

The robust growth in the presence of hemoglobin and hemin led us to investigate the ability of *Paracoccidioides* to internalize protoporphyrin rings. For this assay, the fungus was incubated in the presence or absence of different concentrations of zinc-protoporphyrin IX (Zn-PPIX). The protoporphyrin ring is intrinsically fluorescent, but iron is an efficient quencher of this fluorescence. Consequently, the heme group is not fluorescent, but Zn-PPIX is [61]. Both *Pb*01 and *Pb*18 presented the ability to internalize the protoporphyrin ring because the fluorescence was observed only in fungi that were cultivated with Zn-PPIX (**Figure 2**). The cellular uptake of the compound was concentration- and time- dependent. As the Zn-PPIX concentration increased, the uptake increased in both strains (**Figure 2**). Similarly, increasing the incubation time also enhanced the uptake of Zn-PPIX by both strains (**Figure S1**). To test if another protoporphyrin ring-containing molecule could compete with Zn-PPIX for internalization, a pre-incubation with hemoglobin was performed before incubating the fungus in presence of Zn-PPIX. It was observed that the pre-incubation with hemoglobin, inhibited the Zn-PPIX uptake (**Figure 3**), suggesting that both compounds occupy the same sites for cell internalization. These observations suggest that, to acquire iron from heme, *Paracoccidioides* may internalize the entire molecule to release the iron intracellularly, instead of promoting the iron extraction outside before taking this ion up into cells.

**Figure 4:**
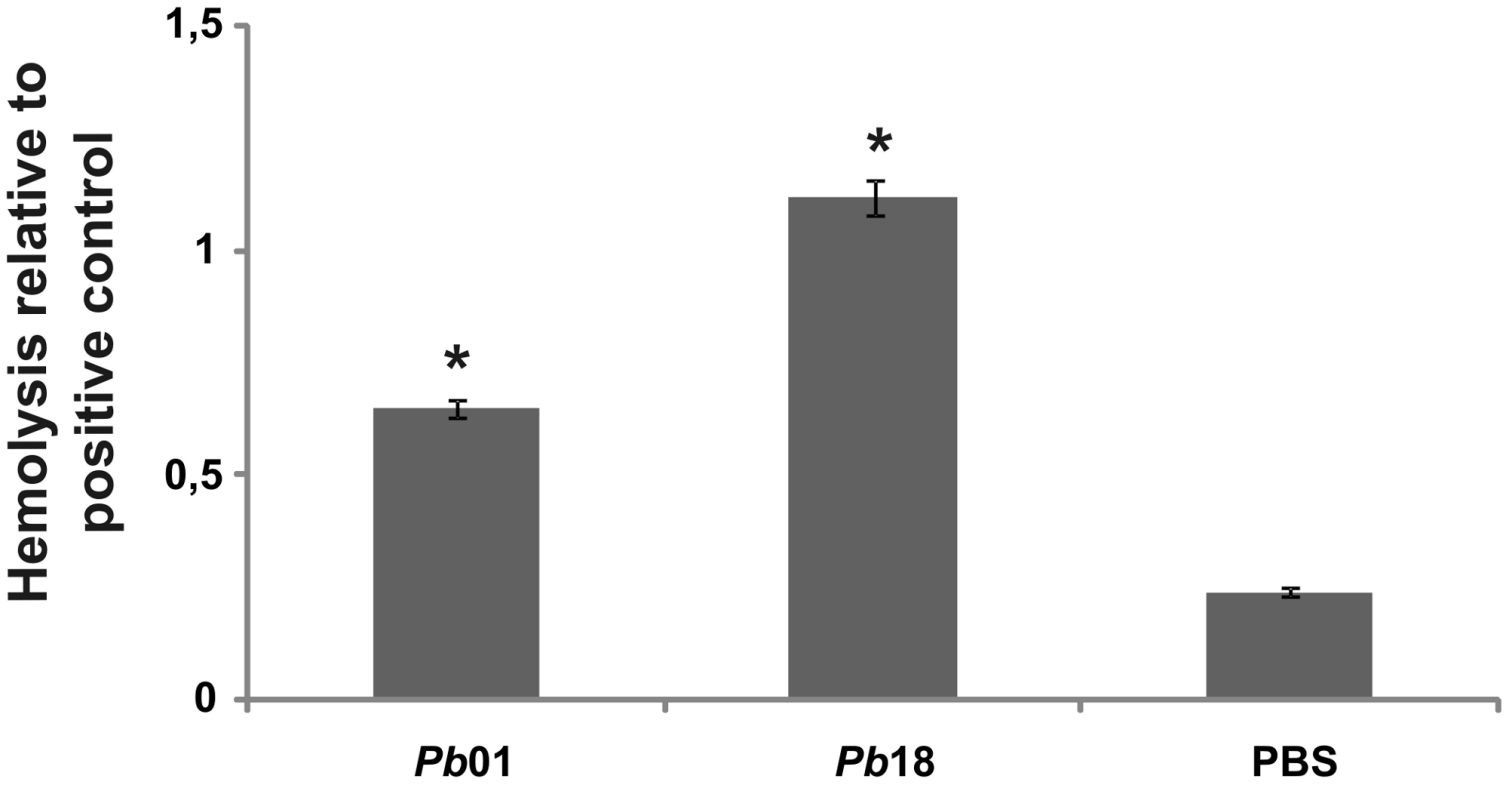
Hemolysis of sheep erythrocytes in the presence of *Paracoccidioides* yeast cells. *Pb*01 and *Pb*18 10^7^ yeast cell suspensions were incubated with 10^8^ sheep erythrocytes for 2 h at 36°C in 5% CO_2_. As a negative or positive control, respectively, erythrocytes were incubated with phosphate buffered saline solution (PBS) or sterile water. The optical densities of the supernatants were determined with an ELISA plate reader at 405 nm. The experiment was performed in triplicate, and the average optical density of each condition was used to calculate the relative hemolysis of the experimental conditions or the negative control against the positive control. The data are plotted as the mean ± standard deviation. *: statistically significant difference in comparison with PBS values according to Student's t-test.

To use hemoglobin as an effective iron source, microorganisms need to lyse host erythrocytes to expose the intracellular hemoglobin. The hemolytic ability of *Paracoccidioides* was assessed by incubating the fungus for 2 hours, after iron starvation, with sheep erythrocytes. Both *Pb*01 and *Pb*18 demonstrated the ability to lyse erythrocytes compared with phosphate buffered saline solution (PBS), which was used as a negative control (**Figure 4**). Sterile water was used as a positive control. Additionally, when *Paracoccidioides* was cultivated in iron presence, the fungus still presented ability to promote erythrocytes lysis (data not shown). These results suggest that *Paracoccidioides* produces a hemolytic factor that can be secreted or that is associated with the fungus surface.

### Transcriptional analysis of *Paracoccidioides* genes that encode putative receptors for hemoglobin uptake

*In silico* searches in the *Paracoccidioides* genome database (http://www.broadinstitute.org/annotation/genome/paracoccidioides_brasiliensis/MultiHome.html) were performed to verify whether the *Pb*01 and *Pb*18 genomes contain genes that encode hemoglobin receptors that are orthologous to genes in the *C. albicans* hemoglobin receptor gene family [19]. The *Pb*01 genome presents three putative hemoglobin receptors that are orthologous to *C. albicans* Rbt5, Wap1/Csa1 and Csa2, respectively. However, *Pb*18 presents only one ortholog to *C. albicans* Wap1/Csa1. Additionally, *Pb*03, which is the other *Paracoccidioides* strain that has its genome published, presents two orthologs, one to *C. albicans* Rbt51 and the other one to *C. albicans* Csa2, as suggested by the *in silico* analysis (**Table S1**). All four putative identified proteins are predicted to have a CFEM domain, which presents eight spaced cysteine residues [22]. These *in silico* analyses suggest that *Paracoccidioides* could uptake hemoglobin through a receptor-mediated process.

**Figure 5:**
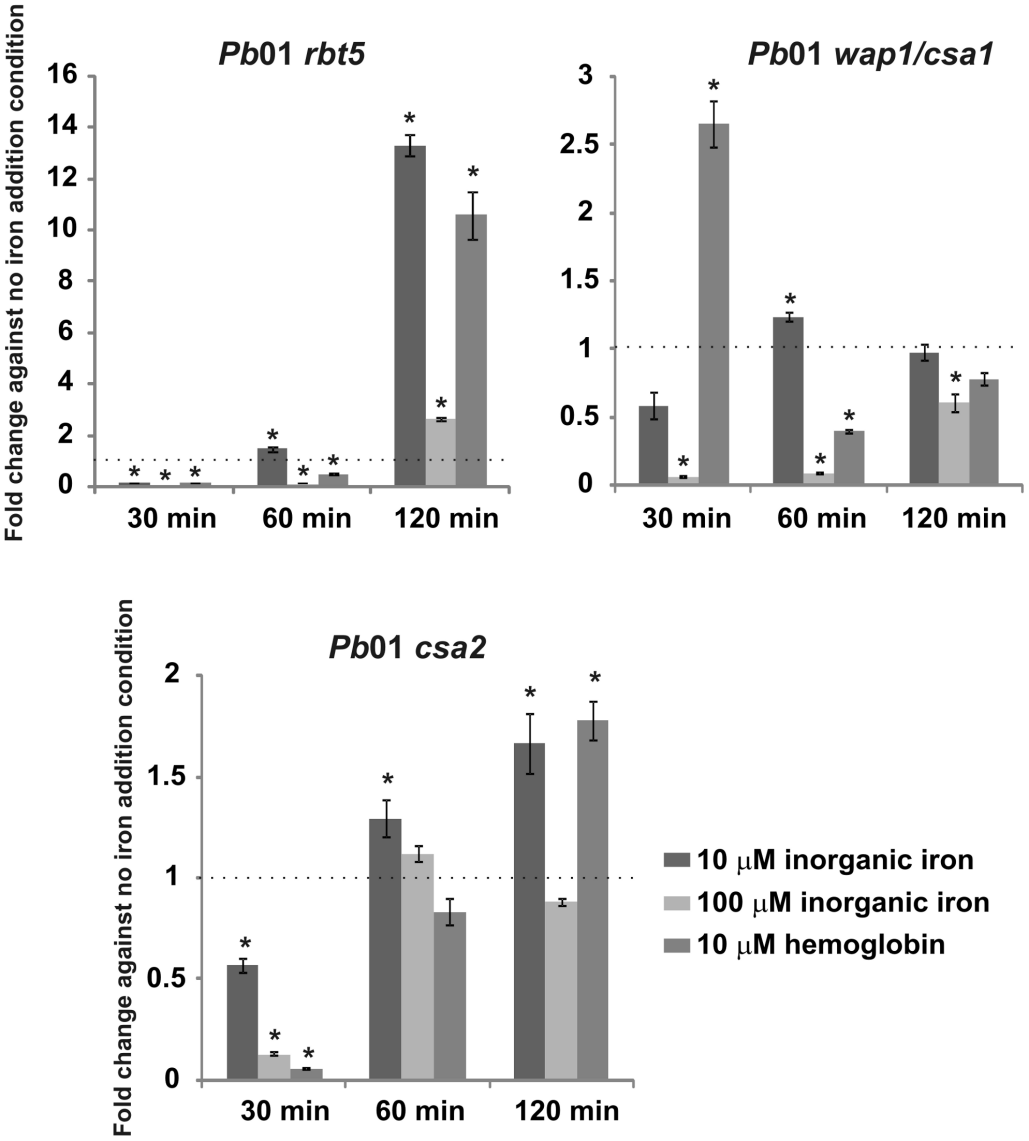
Expression of genes that are putatively related to hemoglobin uptake. *Pb*01 yeast cells were recovered from MMcM medium, which was supplemented or not (no iron addition condition) with different iron sources (10 µM and 100 µM inorganic iron and 10 µM hemoglobin) for 30, 60 and 120 min. After RNA extraction and cDNA synthesis, levels of *Pb*01 *rbt5*, *wap1* and *csa2* transcripts were quantified by qRT-PCR. The expression values were calculated using alpha tubulin as the endogenous control. The values that were plotted on the bar graph were normalized against the expression data that were obtained from the no iron addition condition (fold change). The data are expressed as the mean ± SD of the triplicates. *statistically significant data as determined by Student's t-test (p<0.05).

To analyze the expression of *Pb*01 genes that encode putative hemoglobin receptors, real-time qRT-PCRs were performed with the transcripts that encode the *Pb*01 proteins Rbt5, Wap1/Csa1 and Csa2 in different iron supplementation conditions. As depicted in **Figure 5**, the expression of *Pb*01 putative hemoglobin receptors were regulated at the transcriptional level. One can observe that *rbt5*, *wap1/csa1* and *csa2* transcripts were, in general, up-regulated in iron depletion in comparison to the other conditions tested in this work, at 30 min time point. The expression of *Pb01 rbt5*, for example, increased 20 times in iron-depleted condition in comparison to the presence of 100 µM inorganic iron during 30 min of incubation (data not shown). The exception is *wap1/csa1* in presence of hemoglobin, suggesting that *Pb*01 Wap1/Csa1 might be involved in earlier stages of hemoglobin acquisition. *Pb01 rbt5* was strongly activated after 120 min in the presence of 10 µM inorganic iron or 10 µM hemoglobin, in comparison with the no iron addition condition. This result suggests that *Pb*01 Rbt5 may participate in an iron acquisition pathway that is involved in hemoglobin iron uptake. The *Pb*01 *csa2* transcript presented the same profile, which suggests that *Pb*01 Csa2 may also work in hemoglobin binding/uptake.

### *Paracoccidioides* proteins were induced or repressed in the presence of hemoglobin

Since *Paracoccidioides* appears to use hemoglobin as an iron source, a nanoUPLC-MS^E^-based proteomics approach was employed to identify *Pb*01 proteins that were induced or repressed in the presence of 10 µM hemoglobin as the iron source, compared with 10 µM ferrous ammonium sulfate, which was used as an inorganic iron source. In total, 282 proteins were positively or negatively regulated. In this group, 159 proteins were induced (**Table S2**) and 123 proteins were repressed (**Table S3**) in the presence of hemoglobin, compared with proteins that were produced in the presence of inorganic iron. The false positive rates of protein identification in the presence of hemoglobin and in the presence of inorganic iron were 0.58% and 0.30%, respectively. In total, 75.42% and 76.87% of the peptides that were obtained in the presence of hemoglobin and in the presence of inorganic iron, respectively, were identified in a 5 ppm error range (**Figure S2**). The resulting peptide data that were generated by the PLGS process are shown in **Figure S3**. Some selected proteins that were induced or repressed in the presence of hemoglobin, are depicted in **Tables 1** **and** **2**, respectively. Many proteins that were detected are involved in amino acid, nitrogen and sulfur metabolism (**Tables 1** **and** **2**). Proteins that are involved in alanine, lysine, tryptophan or aspartate and glutamate metabolism, as well as those proteins that are involved in arginine, cysteine, histidine, serine or threonine biosynthesis, were upregulated in the presence of hemoglobin (**Table 1**). In contrast, proteins that are involved in asparagine or phenylalanine degradation were down regulated in the presence of hemoglobin (**Table 2**). This result suggests that the fungus could use hemoglobin not only as iron source, as demonstrated by the induction of proteins that are involved with the iron-sulfur cluster assembly, such as cysteine desulfurase (**Table 1**), but also as nitrogen and sulfur sources because many proteins that are involved in amino acid metabolism were upregulated in the presence of hemoglobin. This observation reinforces the notion that *Paracoccidioides* internalizes the entire hemoglobin molecule instead of promoting the iron release extracellularly. This internalization could occur by endocytosis because proteins that are involved with lysosomal and vacuolar protein degradation, including carboxypeptidase Y and a vacuolar protease A orthologs, were upregulated (**Table 1**). Among the induced proteins, it is important to highlight the *Pb*01 Csa2 detection only in presence of hemoglobin (**Table 1**), which corroborates the hypothesis that hemoglobin uptake by *Paracoccidioides* is receptor-mediated. Among the repressed proteins, those proteins that are involved with porphyrin biosynthesis, including uroporphyrinogen decarboxylase and a glutamate-1-semialdehyde 2,1-aminomutase orthologs, were detected only in the presence of inorganic iron (**Table 2**), which reinforces the hypothesis that hemoglobin is efficiently used by the fungus.

**Table 1:** Relevant *Paracoccidioides Pb*01 proteins that were induced in the presence of hemoglobin as detected by nanoUPLC-MS^E^.

Accession number^a^	Protein description	Score AVG	Peptides AVG	Fold change (Hb:Fe)	E.C. number	Subclassification	Filter^b^
**METABOLISM**							
**Amino acid metabolismo**							
PAAG_02163	acetyl-/propionyl-coenzyme A carboxylase alpha chain	249.65	16.33	***	6.4.1.3	Valine and isoleucine degradation	1
PAAG_07036	methylmalonate-semialdehyde dehydrogenase	279.14	14.83	1.60	1.2.1.27	Valine, leucine and isoleucine degradation	2
PAAG_00221	acetolactate synthase	260.71	10.20	1.55	2.2.1.6	Valine, leucine and isoleucine biosynthesis	2
PAAG_06416	conserved hypothetical protein (alanine racemase)	169.27	4.00	***	5.1.1.1	Alanine metabolism	1
PAAG_08065	aspartate-semialdehyde dehydrogenase	501.59	5.80	3.35	1.2.1.11	Amino acid biosynthesis	2
PAAG_03138	alanine-glyoxylate aminotransferase	381.01	7.33	1.40	2.6.1.44	Amino acid metabolism	2
PAAG_06217	acetylornithine aminotransferase	313.28	11.25	1.72	2.6.1.11	Arginine biosynthesis	1
PAAG_06506	aspartate aminotransferase	253.11	6.50	1.42	2.6.1.1	Aspartate and glutamate metabolism	1
PAAG_06835	cystathionine gamma-lyase	257.79	7.00	***	4.4.1.1	Cysteine biosynthesis	1
PAAG_07813	cysteine synthase	310.64	7.33	***	4.2.1.22	Cysteine biosynthesis	2
PAAG_05392	betaine aldehyde dehydrogenase	913.69	8.67	1.68	1.2.1.8	Glycine biosynthesis	2
PAAG_01568	glycine dehydrogenase	193.28	16.00	***	1.4.4.2	Glycine degradation	2
PAAG_05406	histidine biosynthesis trifunctional protein	196.65	14.00	***	3.5.4.19; 3.6.1.31; 1.1.1.23	Histidine biosynthesis	1
PAAG_00285	imidazole glycerol phosphate synthase hisHF	175.08	19.00	***	2.4.2.-; 4.1.3.-	Histidine biosynthesis	1
PAAG_09095	ATP phosphoribosyltransferase	1303.26	4.67	1.79	2.4.2.17	Histidine biosynthesis	2
PAAG_04099	methylcrotonoyl-CoA carboxylase subunit alpha	179.63	10.00	***	6.4.1.4	Leucine degradation	1
PAAG_06387	homoisocitrate dehydrogenase	431.93	8.00	1.84	1.1.1.87	Lysine biosynthesis	2
PAAG_02693	saccharopine dehydrogenase	241.77	12.67	1.43	1.5.1.10	Lysine metabolism	1
PAAG_07626	cobalamin-independent synthase	1296.09	25.33	2.92	2.1.1.14	Methionine biosynthesis	2
PAAG_06996	G-protein comlpex beta subunit CpcB	1162.11	11.33	2.32	N.A.	Regulation of amino acid metabolism	2
PAAG_03613	phosphoserine aminotransferase	265.03	8.67	4.35	2.6.1.52	Serine biosynthesis	2
PAAG_07760	threonine synthase	171.92	8.00	***	4.2.3.1	Threonine biosynthesis	1
PAAG_08668	anthranilate synthase component 2	242.62	11.50	***	4.1.3.27	Tryptophan biosynthesis	1
PAAG_05005	anthranilate synthase component 1	186.39	14.00	1.38	4.1.3.27	Tryptophan biosynthesis	2
PAAG_02644	kynurenine-oxoglutarate transaminase	179.07	6.00	***	2.6.1.7	Tryptophan degradation	2
PAAG_08164	homogentisate 1,2-dioxygenase	323.07	8.25	1.68	1.13.11.5	Tyrosine degradation	1
**Nitrogen, sulfur and selenium metabolism**							
PAAG_00468	4-aminobutyrate aminotransferase	1947.94	10.50	1.86	2.6.1.19	Nitrogen utilization	2
PAAG_03333	formamidase	14545.80	19.33	1.22	3.5.1.49	Nitrogen compound metabolic process	2
PAAG_05929	sulfate adenylyltransferase	303.58	9.20	2.39	2.7.7.4	Sulfur metabolism	2
**PROTEIN FATE (folding, modification, destination)**							
**Protein/peptide degradation**							
PAAG_02907	conserved hypothetical protein (ankyrin repeat protein)	288.17	6.00	***	N.A.	Cytoplasmic and nuclear protein degradation	1
PAAG_03512	carboxypeptidase Y	496.47	6.33	1.23	3.4.16.5	Lysosomal and vacuolar protein degradation	2
PAAG_01966	hypothetical protein (vacuolar protease A)	299.75	3.33	1.25	3.4.23.25	Lysosomal and vacuolar protein degradation	2
**CELLULAR TRANSPORT, TRANSPORT FACILITIES AND TRANSPORT ROUTES**							
**Transported compounds**							
PAAG_01051	conserved hypothetical protein (*Pb*01 Csa2)	151.83	6.00	***	N.A.	Hemoglobin receptor	1
**INTERACTION WITH THE ENVIRONMENT**							
**Homeostasis**							
PAAG_05851	cysteine desulfurase	160.27	7.00	***	2.8.1.7	Iron-sulfur cluster assembly	1
PAAG_05850	conserved hypothetical protein (cysteine desulfurase)	395.45	10.25	1.65	2.8.1.7	Iron-sulfur cluster assembly	1

a Information that was obtained from the *Paracoccidioides* Database (http://www.broadinstitute.org/annotation/genome/paracoccidioides_brasiliensis/MultiHome.html).

b filter 1 – proteins that were derived from PepFrag2; filter 2 – proteins that were derived from PepFrag1, as determined by PLGS and cited by Murad and Rech (2012).

***: proteins that were identified only in the presence of hemoglobin;

N.A.: not applicable.

**Table 2:** Relevant *Paracoccidioides Pb*01 proteins that were repressed in the presence of hemoglobin as detected by nanoUPLC-MS^E^.

Accession number	Protein description	Score AVG	Peptides AVG	Fold change (Hb:Fe)	E.C. number	Subclassification	Filter
**METABOLISM**							
**Amino acid metabolism**							
PAAG_01206	L-asparaginase	197.88	5.00	***	3.5.1.1	Asparagine degradation	1
PAAG_01365	choline dehydrogenase	206.25	13.00	***	1.1.99.1	Glycine biosynthesis	1
PAAG_02935	glycine cleavage system H protein	404.32	4.33	0.66	N.A.	Glycine degradation	2
PAAG_04102	isovaleryl-CoA dehydrogenase	330.74	7.00	***	1.3.8.4	Leucine degradation	2
PAAG_01974	mitochondrial methylglutaconyl-CoA hydratase	223.92	6.00	***	4.2.1.18	Leucine degradation	1
PAAG_03569	1,2-dihydroxy-3-keto-5-methylthiopentene dioxygenase	175.60	6.00	***	1.13.11.54	Methionine biosynthesis	1
PAAG_08166	4-hydroxyphenylpyruvate dioxygenase	203.44	3.00	***	1.13.11.27	Tyrosine and phenylalanine degradation	1
PAAG_00014	dihydroxy-acid dehydratase	312.52	10.83	0.73	4.2.1.9	Valine and isoleucine biosynthesis	2
PAAG_02554	3-hydroxyisobutyryl-CoA hydrolase	402.87	9.75	0.58	3.1.2.4	Valine degradation	2
PAAG_01194	2-oxoisovalerate dehydrogenase subunit beta	219.86	6.00	***	1.2.4.4	Valine, leucine and isoleucine degradation	2
PAAG_06096	phospho-2-dehydro-3-deoxyheptonate aldolase	1370.44	9.67	0.66	2.5.1.54	Phenyalanine, tyrosine and tryptophan biosynthesis	2
PAAG_07659	chorismate synthase	193.84	3.50	0.61	4.2.3.5	Phenyalanine, tyrosine and tryptophan biosynthesis	2
**Nitrogen, sulfur and selenium metabolism**							
PAAG_04525	glutamine synthetase	156.10	9.00	***	6.3.1.2	Nitrogen metabolism	1
PAAG_07689	NADP-specific glutamate dehydrogenase	2006.41	19.83	0.75	1.4.1.4	Nitrogen metabolism	2
**Secondary metabolism**							
PAAG_00799	uroporphyrinogen decarboxylase	362.04	8.67	***	4.1.1.37	Porphyrin biosynthesis	2
PAAG_06925	conserved hypothetical protein (glutamate-1-semialdehyde 2,1-aminomutase)	322.93	7.00	***	5.4.3.8	Porphyrin biosynthesis	2

a Information that was obtained from the *Paracoccidioides* Database (http://www.broadinstitute.org/annotation/genome/paracoccidioides_brasiliensis/MultiHome.html).

b filter 1 – proteins that were derived from PepFrag2; filter 2 – proteins that were derived from PepFrag1, as determined by PLGS and cited by Murad and Rech (2012).

***: proteins that were identified only in the presence of inorganic iron;

N.A.: not applicable.

### *Paracoccidioides* Rbt5 is a GPI-anchored cell wall protein

We continued our studies with the *Pb*01 ortholog of Rbt5, the best-characterized hemoglobin receptor in *C. albicans* [20]. As described above, *Pb*01 *rbt5* was the transcript that was most efficiently regulated in the presence of hemoglobin. To investigate this result further, a recombinant GST-tagged *Pb*01 Rbt5 protein, which presents 42.5 kDa, was produced in *Escherichia coli* and purified using the GST tag. After purification, the GST tag was removed after thrombin digestion, and the resultant protein presented a molecular mass of 22 kDa (**Figure S4A**). Polyclonal antibodies were raised against the recombinant protein in rabbit. To verify the reactivity of the obtained antibody against the recombinant protein, Western blots were performed (**Figures S4B and S4C**). Only a 22 kDa immunoreactive species was obtained in the Western blot analysis after GST tag cleavage (**Figure S4B, lane 3**). No cross-reactivity was observed with pre-immune sera (**Figure S4C**).

**Figure 6:**
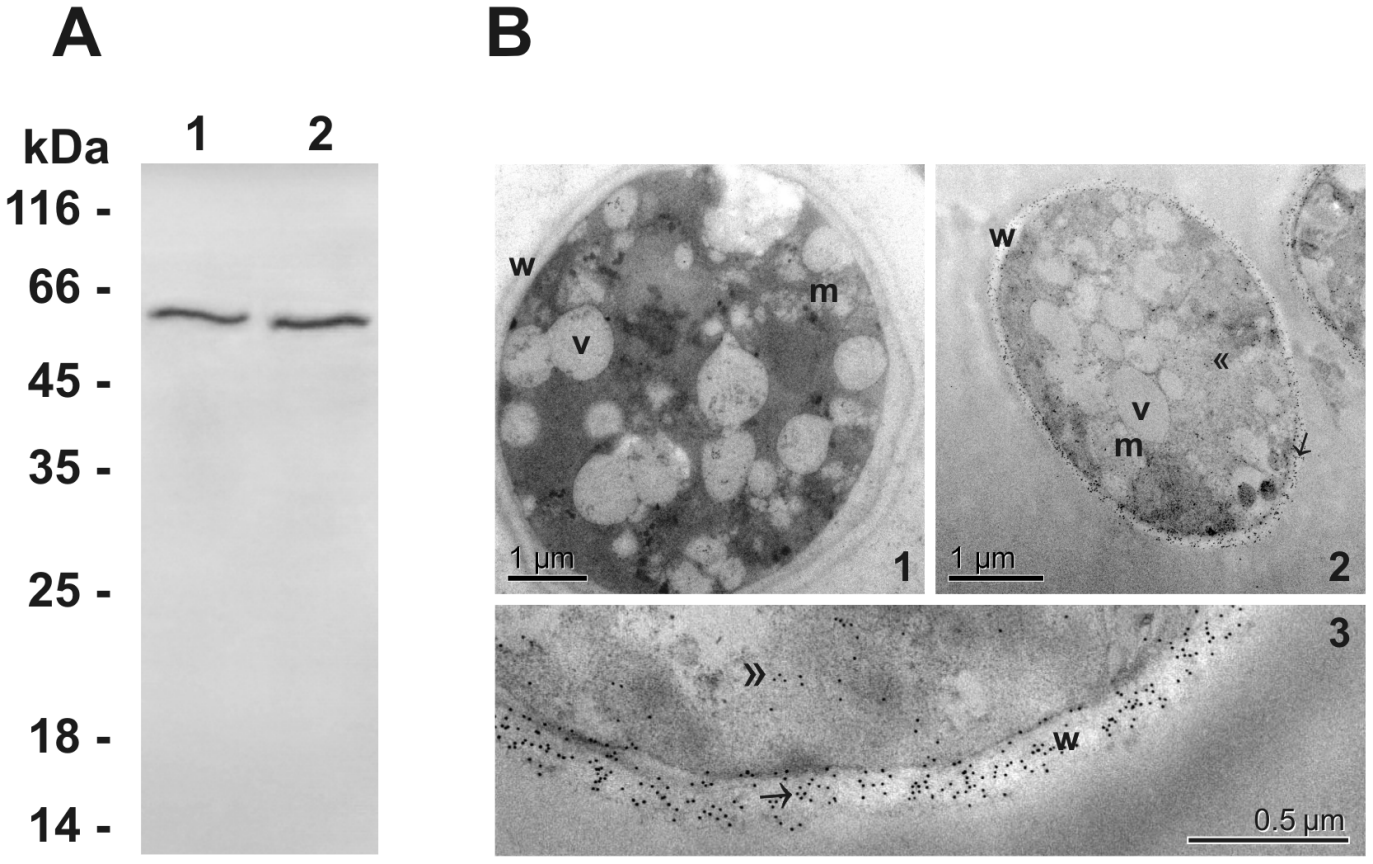
*Paracoccidioides* Rbt5 is a GPI-anchored protein localized in the yeast cell wall. **A**. Cell wall fraction of *Pb*01 yeast cells was obtained and analyzed by Western blot using polyclonal antibodies raised against the recombinant protein Rbt5. Proteins that were obtained from the cell wall (lane 1) were extracted by HF-pyridine digestion and analyzed (lane 2). Molecular weight markers are indicated at the right side of the panel. **B**. Immunoelectron microscopic detection of Rbt5 in *Pb*01 yeast cells by post embedding methods. (1) Negative control exposed to the rabbit preimmune serum. (2 and 3) Gold particles are observed at the fungus cell wall (arrow) and in the cytoplasm (double arrowheads). Bars: 1 µm (1 and 2) and 0.5 µm (3). v: vacuoles. m: mitochondria. w: cell wall.

*In silico* analysis identified a predicted signal peptide and a putative GPI anchor in the *Pb*01 Rbt5 ortholog, which was similar to *C. albicans* Rbt5 (**Figure S5**), suggesting that this protein could localize at the *Paracoccidioides* cell surface. In this way, the GPI-anchored proteins of the *Pb*01 cell wall were extracted using HF-pyridine. A Western blot assay was performed using anti-*Pb*01 Rbt5 polyclonal antibodies against the GPI-anchored protein extract, and a single immunoreactive 60 kDa species was identified in this fraction (**Figure 6A** **, lane 2**). The mass shift from 22 kDa to 60 kDa suggests post-translational modifications (PTMs) of the native protein, which is in agreement with the occurrence of glycosylation [62].

To confirm the cell wall localization, an immunocytochemical analysis of *Pb*01 yeast cells using anti-*Pb*01 Rbt5 polyclonal antibodies was prepared for analysis using transmission electron microscopy (**Figure 6B** **, panels 2 and 3**). Rbt5 was abundantly detected on the *Pb*01 yeast cell wall. Some gold particles were observed in the cytoplasm, which is consistent with intracellular synthesis for further surface export. The control sample was free of label when incubated with the rabbit preimmune serum (**Figure 6B** **, panel 1**).

### *Paracoccidioides* Rbt5 binds heme-containing molecules

**Figure 7:**
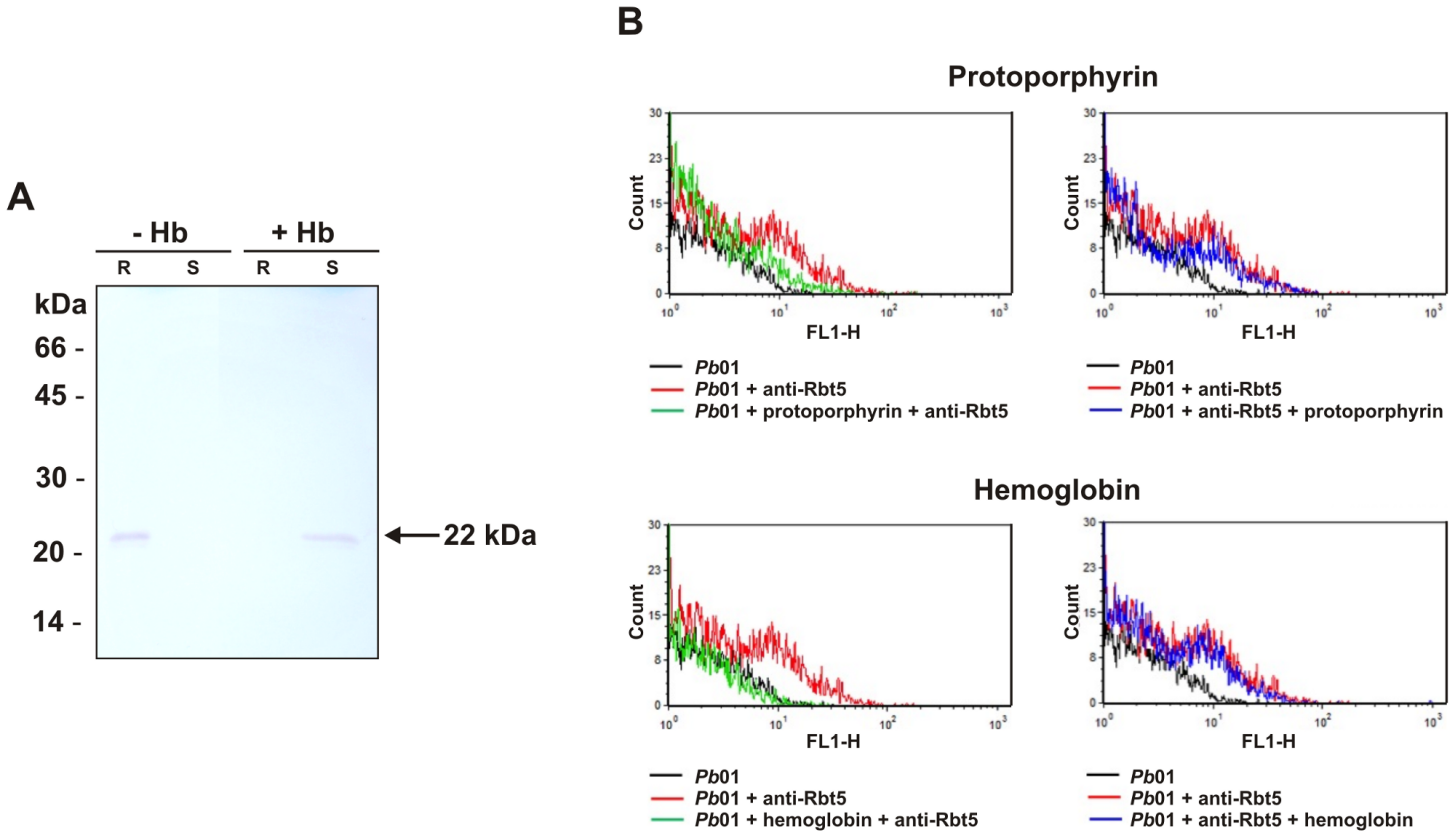
*Paracoccidioides* Rbt5 binds heme-containing molecules. **A**. Recombinant protein Rbt5 was pre-incubated with (+) or without (−) hemoglobin (Hb) for 1 h. Subsequently, the samples were incubated with hemin-agarose resin for 1 h. After, the samples were centrifuged, the supernatants (S) were collected and the resin (R) was washed twice. After adding the buffer, the samples were boiled for 5 min and submitted to SDS-PAGE and Western blot analysis. A single 22 kDa immunoreactive species, which corresponds to the Rbt5 recombinant protein, was detected bound to the resin in the absence of hemoglobin or in the supernatant in the presence of hemoglobin. **B**. Upper and lower panels represent systems where protoporphyrin or hemoglobin Rbt5 recognition was assessed, respectively. The sequential steps of incubation in each system are indicated on the bottom of each panel. *Pb*01 denotes the background fluorescence of fungal cells alone; *Pb*01 + anti-Rbt5 represents systems where fungal cells were sequentially incubated with primary and secondary antibodies; *Pb*01 + protoporphyrin/hemoglobin + anti-Rbt5 is representative of systems that included the blocking of yeast cells with heme-containing molecules before exposure to antibodies; and *Pb*01 + anti-Rbt5 + protoporphyrin/hemoglobin represents systems that included the incubation of yeast cells with heme-containing molecules after exposure to antibodies.

The fact that *Pb*01 Rbt5 is homologous to *C. albicans* Rbt5 (**Figure S5**) suggests that *Pb*01 Rbt5 may participate in hemoglobin uptake in *Paracoccidioides*. In this way, the protein's ability to interact with the heme group was investigated. Affinity assays were performed using the recombinant *Pb*01 Rbt5 and a hemin-agarose resin (**Figure 7A**). A specific ability to interact with hemin was demonstrated to *Pb*01 Rbt5, since enolase, which is also present at the *Pb*01 yeast surface [52], did not present ability to bind to the hemin resin (data not shown). Moreover, when pre-incubating the recombinant Rbt5 with hemoglobin, the Rbt5 was not able anymore to interact with the hemin resin, suggesting that hemoglobin compete with hemin for the Rbt5 binding sites (**Figure 7A**).

To confirm the ability of Rbt5 to recognize heme-containing molecules, *Pb*01 yeast cells were submitted to binding assays for further flow cytometry analyses. Background fluorescence levels were determined using yeast cells alone (**Figure 7B** **, black lines**). Positive controls were composed of systems where *Pb*01 cells were incubated with polyclonal antibodies raised against *Pb*Rbt5, followed by incubation with a fluorescent secondary antibody (**Figure 7B** **, red lines**). For the determination of binding activities, *Pb*01 yeast cells were incubated with protoporphyrin or hemoglobin before or after exposure to the anti-*Pb*Rbt5 antibodies, followed by incubation with the secondary antibody (**Figure 7B** **, green and blue lines, respectively**). When yeast cells were incubated with protoporphyrin or hemoglobin before exposure to primary and secondary antibodies, fluorescence intensities were at background levels, suggesting that the heme-containing molecules blocked surface sites that are also recognized by the anti-*Pb*Rbt5 antibodies (**Figure 7B**). When the cells were exposed to protoporphyrin or to hemoglobin after incubation with the antibodies, the fluorescence levels were similar to those levels that were obtained in systems where incubation with the heme-containing proteins was omitted. These results suggest a high-affinity binding between Rbt5 and heme-containing molecules, which corroborates the hypothesis that Rbt5 could act as a hemoglobin receptor at the fungus cell surface.

### *Paracoccidioides rbt5* knockdown decreases survival inside the host

**Figure 8:**
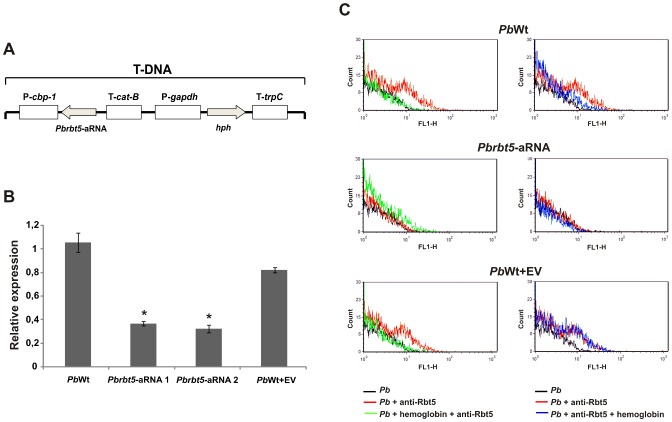
*Paracoccidioides* Rbt5 knock down via an antisense-RNA (aRNA) strategy. **A**. Schematic representation of the T-DNA cassette that was used in this work to perform the *Agrobacterium tumefaciens*-mediated transformation (ATMT) of *Pb*339 (*Pb*Wt). *Pbrbt5*-aRNA was cloned in the pUR5750 binary vector under the control of the *Histoplasma capsulatum cbp-1* gene promoter region (P-*cbp-1*) and the *Aspergillus fumigatus cat-B* gene termination region (T-*cat-B*). The selection marker that was used in this work was the *Escherichia coli* hygromycin-resistance gene *hph*. In the cassette, this gene is flanked by the glyceraldehyde-3-phosphate dehydrogenase promoter region (P-*gapdh*) and by the *trpC* termination region (T-*trpC*) from *Aspergillus nidulans*. **B**. After the selection of mitotic stable isolates, a qRT-PCR was performed to analyze the silencing level of the gene in isolates that were transformed with *Pbrbt5*-aRNA. As controls, *rbt5* transcript level from *Pb*Wt and *Pb*Wt transformed with the empty vector (*Pb*Wt+EV) were also quantified. Alpha tubulin was used as the endogenous control. The data are represented as the means ± SD from triplicate determinations. *: statistically significant data as determined by Student's t-test (p<0.05) in comparison with the data that were obtained from *Pb*Wt+EV strain. **C**. Effect of *Pb*rbt5 deletion on the interaction of *Paracoccidioides* with heme-containing molecules. Hemoglobin prevents *Pb*Wt and *Pb*Wt+EV cells to be recognized by the anti-Rbt5 antibodies. However, *Pb*rbt5-aRNA cells are poorly recognized by the antibody that was raised against Rbt5, which is a process that was not affected by the previous or subsequent exposure of yeast cells to hemoglobin.

To verify whether Rbt5 deficiency could influence the ability of the fungus to acquire heme groups or to survive inside the host, an antisense-RNA (aRNA) strategy was applied (**Figure 8A**). For this analysis, *Pb*339 was used, since the *Agrobacterium tumefaciens*-mediated transformation (ATMT) of this strain has been standardized [63]. The knockdown strategy was demonstrated to be efficient because the quantification of *rbt5* transcripts in two isolates of knockdown strain (*Pbrbt5*-aRNA 1 and *Pbrbt5*-aRNA 2) was 60% lower than in the wild type strain (*Pb*Wt) (**Figure 8B**). The strain that was transformed with the empty vector (*Pb*Wt+EV) showed a similar level of *rbt5* transcripts compared with *Pb*Wt (**Figure 8B**). Because of its higher stability, the *Pbrbt5*-aRNA 1 isolate was selected for the next experiments. The flow cytometry results with *Pb*Wt and *Pb*Wt+EV strains (**Figure 8C**) were similar to those results that are described in **Figure 7B**. In contrast, fluorescence intensities were all at background levels when the *Pb*rbt5-aRNA strain was assessed. These results indicate that the gene silencing was efficient also at protein level.

Despite the efficiency of the knockdown strategy, the *Pbrbt5*-aRNA strain demonstrated an identical ability to grow in the presence of hemoglobin as the iron source, compared to the other strains (**Figure S6**), which suggests that either a low amount of Rbt5 at the cell surface is sufficient to allow hemoglobin acquisition or that the other putative hemoglobin receptors could compensate for the Rbt5 deficiency. The identical growth ability of all three strains was also observed in media without iron and with ferrous ammonium sulfate as an inorganic iron source (**Figure S6**). However, the incubation in presence of Zn-PPIX demonstrated a decreased fluorescence of the *Pbrbt5*-aRNA strain in comparison to the other control strains (**Figure S7**), corroborating the hypothesis that *Paracoccidioides* Rbt5 could function as a hemoglobin receptor at the cell surface.

**Figure 9:**
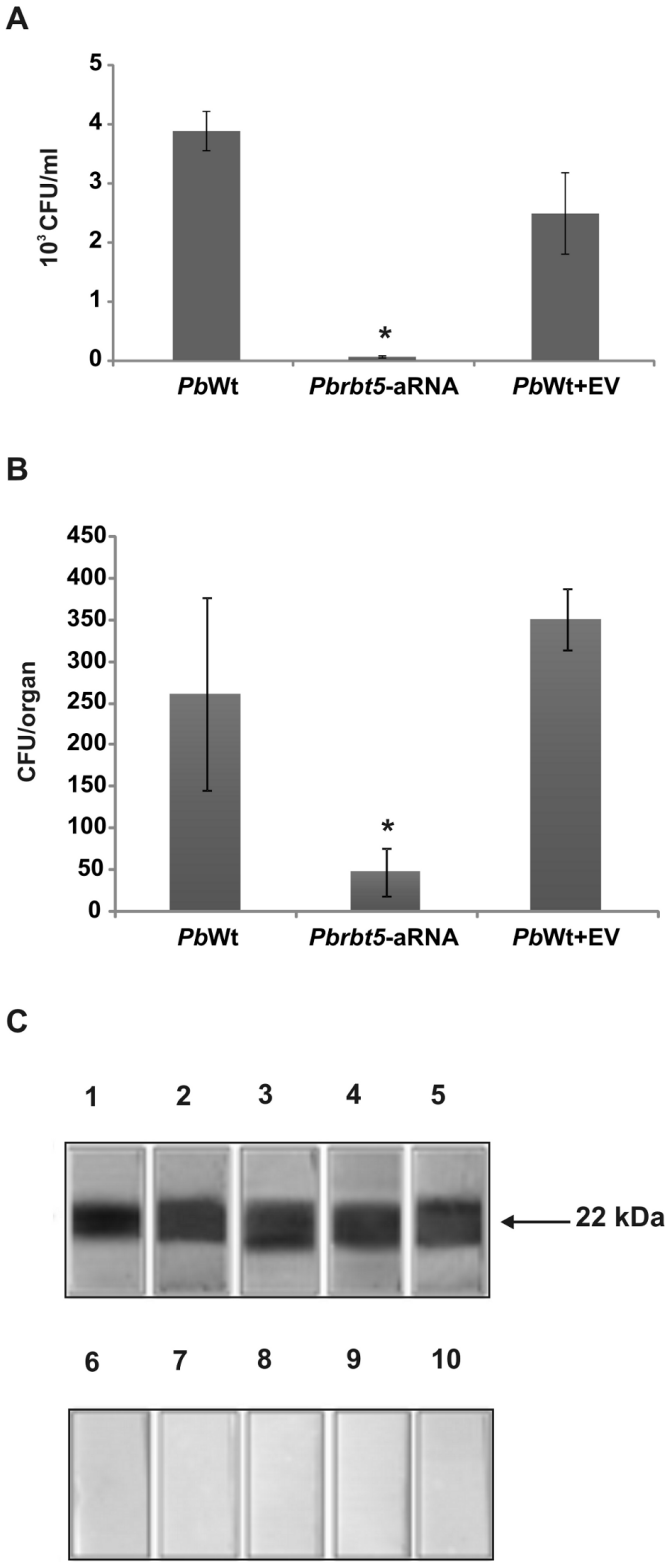
*Paracoccidioides* Rbt5 shows virulent and antigenic properties. **A**. To test the ability to infect macrophages, *Pb*Wt, *Pbrbt5*-aRNA or *Pb*Wt+EV strains were co-cultivated with macrophages for 24 h. After this period, infected macrophages were lysed, and lysates were plated on BHI medium to recover the fungi. The data are presented as a bar graph of the means ± SEM from triplicates. *: statistically significant data as determined by Student's t-test (p<0.05) in comparison with the data that were obtained from the *Pb*Wt+EV strain. **B**. A murine model of infection was also used. Mice were infected intraperitoneally with *Pb*Wt, *Pbrbt5*-aRNA or *Pb*Wt+EV strains. After 2 weeks of infection, mice were sacrificed, the spleens were removed and samples of the homogenate were plated on BHI medium. After 15 days, the CFUs were counted to determine the fungal burden for each strain. The data are presented as a bar graph of the means ± SEM from quadruplicates. *: statistically significant data as determined by Student's t-test (p<0.05) in comparison with the data that were obtained from the *Pb*Wt+EV strain. **C**. Reaction of the recombinant *Pb*01 Rbt5 with sera of five PCM patients (lanes 1–5) or with control sera (lanes 6–10). After reacting with the anti-human IgG peroxidase coupled antibody, the reaction was developed using hydrogen peroxide and diaminobenzidine.

To test the ability of *Paracoccidioides* mutant strains to survive inside the host, two strategies were employed. First, *Pbrbt5*-aRNA and *Pb*Wt+EV were co-cultivated with macrophages. *Pb*Wt was used as a control. After 24 h, macrophages were first washed with PBS to remove the weakly bounded yeast cells and then were lysed with distilled water. Lysates were plated on BHI solid medium to recover the internalized fungi. After 10 days, the colony forming units (CFUs) were counted, and the *Pbrbt5*-aRNA presented approximately 98% reduction in the number of CFUs in comparison with *Pb*Wt and *Pb*Wt+EV (**Figure 9A**). The second strategy included a murine model of infection. Mice were inoculated intraperitoneally with *Pb*Wt, *Pb*Wt+EV and *Pbrbt5*-aRNA, independently. After 2 weeks of infection, the mice were sacrificed, and the spleens were removed. The organs were macerated, and the homogenized sample was plated on BHI agar for CFU determination. The number of CFUs after the infection with the *Pbrbt5*-aRNA strain was approximately 6 times lower than the CFUs that were observed after the infection with *Pb*Wt or with *Pb*Wt+EV (**Figure 9B**). These results indicate that the *rbt5* knockdown could reduce the virulence of the fungi and/or increase the stimulation of the host defense cells to kill the fungus.

To verify whether *Pb*Rbt5 had antigenic properties, sera of five PCM patients were used in immunoblot assays against the recombinant protein. All sera presented strong reactivity against the recombinant *Pb*01 Rbt5 that was immobilized in the nitrocellulose membrane (**Figure 9C** **, lanes 1–5**). No cross-reactivity was observed with control sera of patients who were not diagnosed with PCM (**Figure 9C** **, lanes 6–10**). This result suggests that *Pb*01 Rbt5 is an antigenic protein that is produced by *Paracoccidioides* during human infection.

## Discussion

Because pathogenic fungi face iron deprivation in the host, these microorganisms have evolved different mechanisms to acquire iron from the host's iron-binding proteins [64]. *C. albicans*, for example, can use transferrin, ferritin and hemoglobin as host iron sources [15], [16], [19], [20]. It has been demonstrated in *Paracoccidioides* that genes that are involved in iron acquisition are not upregulated during the incubation of the fungus with human blood, which suggests that this condition is not iron-limiting for this fungus [60]. This observation, coupled with the *Paracoccidioides* preference for heme iron in culture, suggests heme iron scavenging during infection.

In this study, we observed that *Paracoccidioides* presented the ability to internalize a zinc-bound protoporphyrin ring in a dose- and time- dependent pattern. It seems that hemoglobin and Zn-PPIX occupy the same receptor sites, since hemoglobin blocked Zn-PPIX internalization. Moreover, the fungus could promote erythrocyte lysis. A hemolysin-like protein (XP_002797334) has been evidenced in a mycelium to yeast transition cDNA library [65], which indicates that *Paracoccidioides* could access the intracellular heme in the host by producing a hemolytic factor that can be secreted or associated with the fungus surface. The ability to internalize the zinc-bound protoporphyrin ring has been demonstrated for *C. albicans*, but not for *C. glabrata* [61]. The absence of protoporphyrin internalization by *C. glabrata* is most likely because heme receptors are not present in this fungus, as suggested by the fact that genes that encode these receptors have not been identified in the *C. glabrata* genome [61]. In contrast, a hemoglobin-receptor gene family that is composed of the genes *rbt5*, *rbt51*, *wap1/csa1*, *csa2* and *pga7* has been identified in *C. albicans* [19]. To access the heme group inside the erythrocytes, *C. albicans* also produces a hemolytic factor that is able to promote the lysis of erythrocytes [17].

By performing an *in silico* analysis, iron-related genes were identified in the *Paracoccidioides* genome, which were composed of *rbt5*, *wap1/csa1* and *csa2*, that were orthologous to *C. albicans* genes that encode hemoglobin receptors [19], providing further evidence that *Paracoccidioides* has the ability to use hemoglobin in its regular metabolic pathways. In *C. albicans*, the transcripts *rbt5* and *wap1* are activated during low iron condition compared with high iron abundance conditions [24], which corroborate the hypothesis that these transcripts are involved in an iron acquisition mechanism, more specifically, in hemoglobin uptake. Similar results were obtained with *Paracoccidioides*. The *rbt5*, *wap1/csa1* and *csa2* transcripts were also induced in the fungus *Pb*01. Moreover, most of these transcripts were induced in a low-inorganic iron condition or in the presence of hemoglobin compared with iron depletion, after 30 minutes of incubation. These results suggest that the proteins that are encoded by the analyzed transcripts could be involved in hemoglobin utilization in *Pb*01.

The proteomic analysis of the *Pb*01 strain demonstrated that Csa2 was detected only in the presence of hemoglobin, which suggests that its uptake by *Paracoccidioides* is receptor-mediated, as described for *C. albicans* [19] and *C. neoformans* [26]. Among the three *Pb*01 hemoglobin-receptor orthologs, Csa2 is the only one that was not predicted to have a GPI-anchor (**Table S1**). Because no specific protocol to purify GPI-surface proteins has been used for proteomic analyses, no additional hemoglobin-binding proteins than Csa2 were identified in this proteome. The *Pb*01 proteome in the presence of hemoglobin demonstrated that proteins that are involved in amino acid, nitrogen and sulfur metabolism and in iron-sulfur cluster assembly were induced in comparison with the fungus that was cultivated in presence of inorganic iron. Moreover, proteins that are involved in porphyrin biosynthesis were detected only when the fungus was cultivated in the presence of inorganic iron. These results suggest that the fungus could use hemoglobin as an efficient source of nitrogen, sulfur, iron and porphyrin, internalizing the entire hemoglobin molecule. This internalization hypothesis is corroborated by the fact that proteins that are involved with lysosomal and vacuolar protein degradation were also induced in the presence of hemoglobin. Similar mechanisms have been suggested for *C. albicans* and *C. neoformans*. In *C. albicans*, hemoglobin is taken up by endocytosis after Rbt5/51 binding [20]. In *C. neoformans*, Cig1, a recently described extracellular mannoprotein that functions as a receptor or hemophore at the cell surface [26], potentially helps the fungus to take up heme before iron release, perhaps by endocytosis [27].

*Pb*01 *rbt5* presented a high level of transcriptional regulation in the presence of hemoglobin, as observed in this work. In this way, *Pb*01 Rbt5 was investigated and demonstrated characteristics that were similar to *C. albicans* Rbt5, such as the presence of a CFEM domain [9] and a GPI anchor [66]. *Pb*01 Rbt5 was identified in the cell wall extract, which was enriched with GPI proteins, obtained as previously described [49], and was visualized at the *Pb*01 yeast surface. These results indicate that *Pb*01 Rbt5 is anchored at the fungal surface through a GPI anchor. To function as a hemoglobin receptor, a protein must be able to bind heme. It has been suggested that the CFEM domain is able to bind to ferrous and ferric iron, including the iron atom present in the center of the heme group [23], suggesting that *Pb*01 Rbt5 potentially binds to heme group. *Pb*01 Rbt5 heme group-binding ability and the competition between hemoglobin and hemin for the same *Pb*01 Rbt5 binding sites were demonstrated using batch ligand affinity chromatography, with a hemin-resin and the *Pb*01 Rbt5 recombinant protein. Moreover, flow cytometry assays using the whole *Pb*01 yeast cells and the anti-Rbt5 antibodies, which were raised against the *Pb*01 Rbt5 recombinant protein, in the presence of protoporphyrin and hemoglobin also demonstrated the *Pb*01 Rbt5 affinity for these two heme-containing molecules. In *C. neoformans*, the Cig1 heme-binding ability was detected using spectrophotometric titration and isothermal titration calorimetry assays with recombinant Cig1-GST protein purified from *E. coli* [26]. These results demonstrated that *Pb*01 Rbt5 is able to bind hemin, protoporphyrin and hemoglobin, which corroborate the hypothesis that *Pb*01 Rbt5 could function as a heme group receptor, which could help in the acquisition of iron from host sources.

Functional genomic studies in *Paracoccidioides* are recent because little is known regarding the fungi's life cycle. For instance, mechanisms of homologous recombination or haploid segregation in *Paracoccidioides* cells remain obscure. This paucity of data compromises the development of efficient classical genetic techniques [56]. Thus, to modulate the expression of target genes in *Paracoccidioides*, antisense RNA (aRNA) technology is applicable by *A. tumefaciens*-mediated transformation (ATMT) [53], [56], [63]. In this work, *Paracoccidioides rbt5* knockdown strains were generated using the same methodologies. Reductions in gene and protein expression in *Pbrbt5*-aRNA strains were demonstrated by qRT-PCR and flow cytometry assays, respectively, in comparison with the control strains (wild type and empty vector transformed strains). It was also observed a reduced ability of the knock down strain to uptake heme groups, as demonstrated by the decreased Zn-PPIX internalization. Despite the knockdown of *Pb*Rbt5, no growth difference was observed in the presence of inorganic iron or hemoglobin sources. In contrast, in *C. albicans*, a *Δrbt5* mutant strain presented reduced growth in the presence of hemin and hemoglobin as iron sources [19]. These results suggest that other hemoglobin receptors could function at the *Paracoccidioides* surface; this possibility is the focus of future studies in our laboratory.

*Paracoccidioides* is a thermodimorphic fungus that can infect the host by airborne propagules. After the mycelium-yeast transition in the host lungs, the fungus can disseminate to different organs and tissues through the hematogenous or lymphatic pathways [67], [68]. In the host tissues, including the lungs, the fungus can be internalized by macrophages [31], [35]. One of the functions of the macrophages is to recycle senescent red cell iron, primarily in the spleen. Hemoglobin-derived heme is catabolized and the heme iron is released by a hemoxygenase inside macrophages [5]. In this way, *Paracoccidioides* has at least two different opportunities to be exposed to the heme group: during (i) fungal dissemination by the hematogenous route or (ii) macrophage infection. Because it has been suggested that monocyte intracellular iron availability is required for *Paracoccidioides* survival [37], the ability of the *rbt5* knockdown strain to survive inside macrophages was investigated. The *rbt5* knockdown strain presented decreased survival inside macrophages in comparison with control strains, which indicates that Rbt5 could be a virulence factor and/or could affect macrophage stimulation to kill the internalized yeast cells. In addition, the fungal burden in mouse spleen that was infected with the *rbt5* knockdown strain was lower than the fungal burden of the mice that were infected with the control strains, indicating that Rbt5 could be important for infection establishment and/or maintenance by *Paracoccidioides*. The differences observed between the *in vitro* and *in vivo* conditions may be due to host defense against *Paracoccidioides* in animals and macrophage. In contrast, the *rbt5* deletion did not affect *C. albicans* virulence in animal models of infection [25], which indicates that other compensatory mechanisms could act in the absence of Rbt5 in this fungus [19]. The ability of Rbt5 to function as an antigen in *Paracoccidioides* was demonstrated by *Pb*01 Rbt5 recombinant protein recognition using sera of five PCM patients in immunoblot assays. Similar results were obtained for *C. albicans* because Rbt5 and Csa1 were found among 33 antigens that were recognized by sera from convalescent candidemia patients [69]. These results reinforce that Rbt5 could act in the host-pathogen interface.

Fungal surface proteins that are involved in iron uptake might be attractive targets for vaccines or drugs that block microbial proliferation. Moreover, these proteins could be considered as routes to introduce antifungal agents into fungal cells [70]. In that way, iron acquisition mechanisms could be important targets to prevent or treat fungal diseases. This study constitutes evidence that *Paracoccidioides* could acquire heme groups through a receptor-mediated mechanism. In that way, Rbt5 may be a good target for developing vaccines, for blocking *Paracoccidioides* proliferation inside phagocytes, or for using a Trojan horse strategy for introducing antifungal agents into yeast cells.

## Supporting Information

Figure S1 **Zn-PPIX acquisition by** ***Paracoccidioides*** **is time-dependent.** Iron deprived *Pb*01 and *Pb*18 yeast cells were incubated in MMcM medium supplemented with 60 µM zinc protoporphyrin IX (Zn-PPIX) for different times (0–10 h). After those periods, the cells were washed twice, and observed by bright field microscopy (BF) and by live fluorescence microscopy (F).
(TIF)

Figure S2 **Peptide error level that was obtained via the nanoUPLC-MS^E^ approach.** The peptide and protein tables from PLGS were analyzed using the Spotfire software, which generated the ppm error graphics. These graphics indicate the number of peptides in a 15 ppm error range that were either obtained in the presence of 10 µM hemoglobin or obtained in the presence of 10 µM inorganic iron.
(TIF)

Figure S3 **Peptide detection type that was used by the PLGS software.** PLGS software uses an iterative search strategy for peptide identification as described previously [71]. During the first iteration (PepFrag1), only completely cleaved tryptic peptides are used for identification. The second pass of the database algorithm (PepFrag2) is designed to identify peptide modifications and nonspecific cleavage products to proteins that were positively identified in the first pass. VarMod: variable modifications. InSource: fragmentation that occurred on ionization source. MissedCleavage: missed cleavage performed by trypsin. NeutralLoss H_2_O and NH_3_ correspond to water and ammonia precursor losses. The Spotfire software was used to generate the graphics.
(TIF)

Figure S4 **Expression and purification of the** ***Pb*** **01 recombinant Rbt5 and the generation of a rabbit polyclonal antibody.** **A**. SDS-PAGE analysis of *Pb*01 recombinant Rbt5. *E. coli* cells harboring the pGEX-4T-3-Rbt5 plasmid were grown to an OD_600_ of 0.8 and harvested before (lane 1) or after (lane 2) incubation with IPTG. The cells were lysed by sonication, the recombinant protein was purified (lane 3) and the fusion protein (glutathione S-transferase, GST) was cleaved by thrombin digestion (lane 4). Electrophoresis was performed on 10% SDS–PAGE, and the proteins were stained by Coomassie blue R-250. **B and C**. Western blot analysis of the recombinant Rbt5. The proteins that were obtained were screened using the rabbit polyclonal antibody anti-rRbt5 (**B**) or the rabbit preimmune serum (**C**). In B and C: *E. coli* C41 (DE3) that were transformed with the PGEX-4T-3-Rbt5 construct protein extract (lane 1); the affinity-isolated recombinant GST-Rbt5 (lane 2); the recombinant fusion protein cleaved with thrombin (lane 3). The reaction was developed using BCIP-NBT. Arrows indicate the deduced molecular mass of the proteins. Molecular markers are indicated at the left side of the panels.
(TIF)

Figure S5 ***Pb*** **01 Rbt5 and** ***Candida*** **Rbt5 alignment.** The amino acid sequences of the orthologs were aligned using the software ClustalX2. Asterisks: amino acid identity. Dots: conserved substitutions. In bold: signal peptide predicted by SignalP 4.1 Server. Grey box: CFEM domain that was predicted by the SMART online tool. In italic: cysteine residues inside the CFEM domain. Black border rectangle: omega-site that was predicted by the big-PI Fungal Predictor online tool.
(TIF)

Figure S6 ***Paracoccidioides rbt5*** **knock down did not affect fungus growth under different iron availability conditions.** *Pb*339 (*Pb*Wt), the *rbt5* knock down strain (*Pb*rbt5-aRNA) and the *Pb*339 strain that was transformed with the pUR5750 empty vector (*Pb*Wt+EV) were collected after 36 h of iron scarcity, washed, and ten-fold serial dilutions of cell suspensions (10^5^ to 10^2^ cells) were spotted on MMcM medium plates that were supplemented with 50 µM BPS, which is an iron chelator. As indicated, 30 µM inorganic iron or 30 µM hemoglobin were added or not (no iron).
(TIF)

Figure S7 ***Paracoccidioides rbt5*** **knock down strain presents reduced Zn-PPIX uptake.** Iron deprived *Pb*339 (*Pb*Wt), *rbt5* knock down strain (*Pb*rbt5-aRNA) and *Pb*339 strain that was transformed with the pUR5750 empty vector (*Pb*Wt+EV) cells were incubated in MMcM medium supplemented (+) or not (−) with 60 µM zinc protoporphyrin IX (Zn-PPIX) for 2 h. After this period, the cells were washed twice, and observed by bright field microscopy (BF) and by live fluorescence microscopy (F).
(TIF)

Table S1 **Predicted members of the hemoglobin-receptor gene family in** ***Paracoccidioides*** **genus.**
(DOCX)

Table S2 ***Paracoccidioides Pb*** **01 proteins induced in presence of hemoglobin.**
(DOCX)

Table S3 ***Paracoccidioides Pb*** **01 proteins repressed in presence of hemoglobin.**
(DOCX)
