# Supplementary figures and images for: Hemoglobin Uptake by Paracoccidioides spp. Is Receptor-Mediated
Source: PLoS Negl Trop Dis. 2014 May 15;8(5):e2856. doi: 10.1371/journal.pntd.0002856 (PMC4022528; doi:10.1371/journal.pntd.0002856)

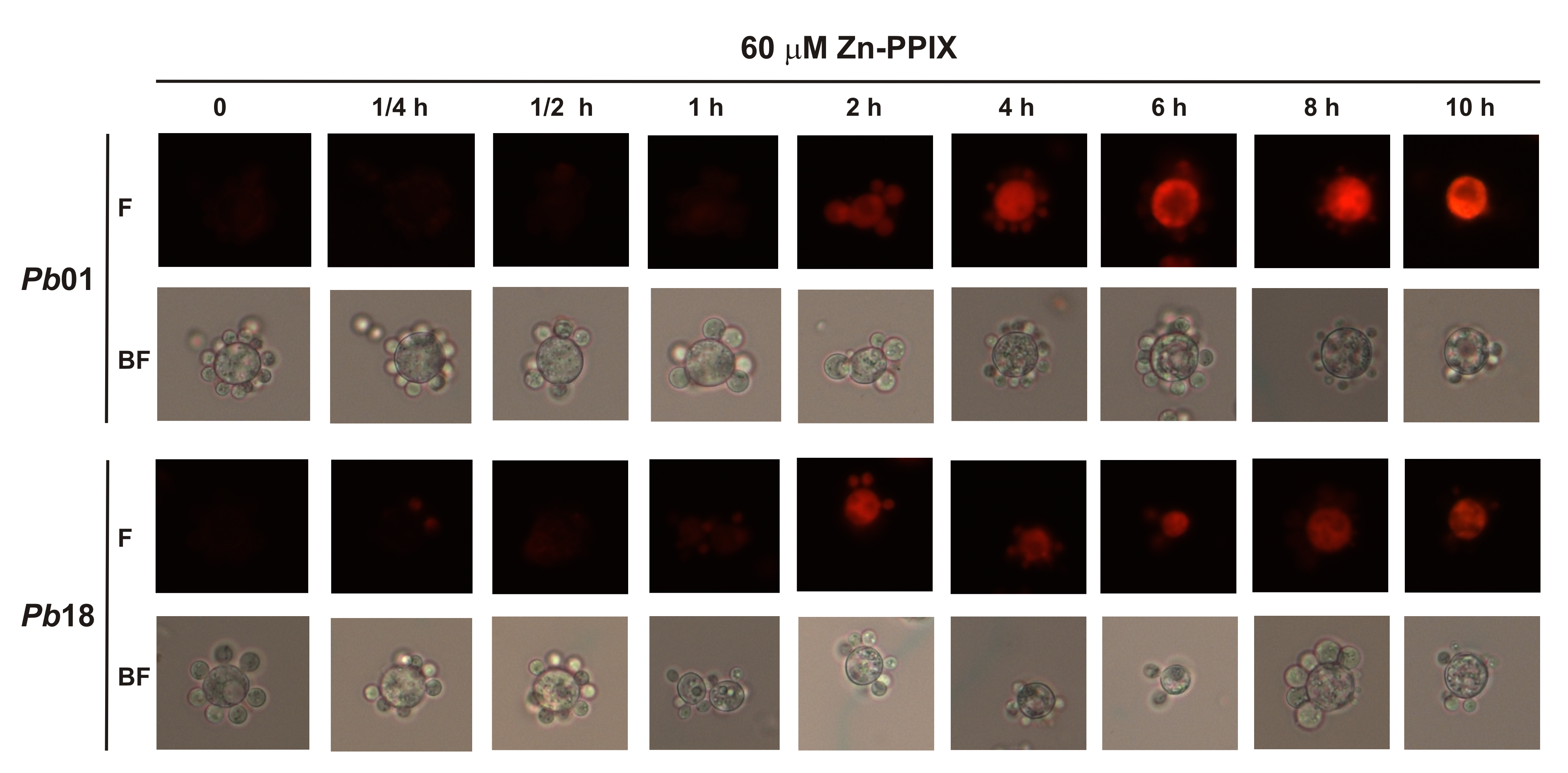

Supplement: Figure S1 — Zn-PPIX acquisition by Paracoccidioides is time-dependent. Iron deprived Pb01 and Pb18 yeast cells were incubated in MMcM medium supplemented with 60 µM zinc protoporphyrin IX (Zn-PPIX) for different times (0–10 h). After those periods, the cells were washed twice, and observed by bright field microscopy (BF) and by live fluorescence microscopy (F). (TIF) [file pntd.0002856.s001.tif]

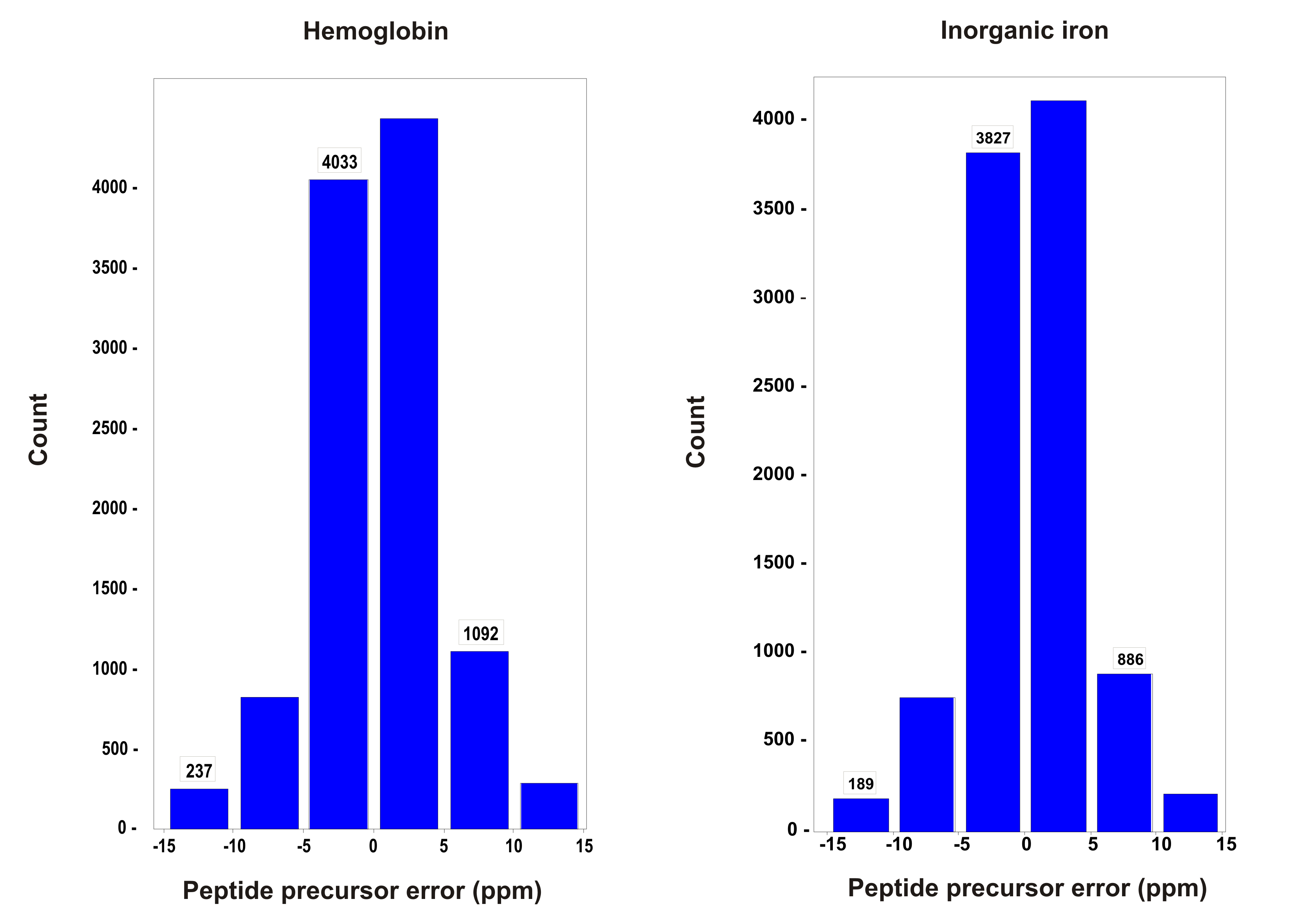

Supplement: Figure S2 — Peptide error level that was obtained via the nanoUPLC-MSE approach. The peptide and protein tables from PLGS were analyzed using the Spotfire software, which generated the ppm error graphics. These graphics indicate the number of peptides in a 15 ppm error range that were either obtained in the presence of 10 µM hemoglobin or obtained in the presence of 10 µM inorganic iron. (TIF) [file pntd.0002856.s002.tif]

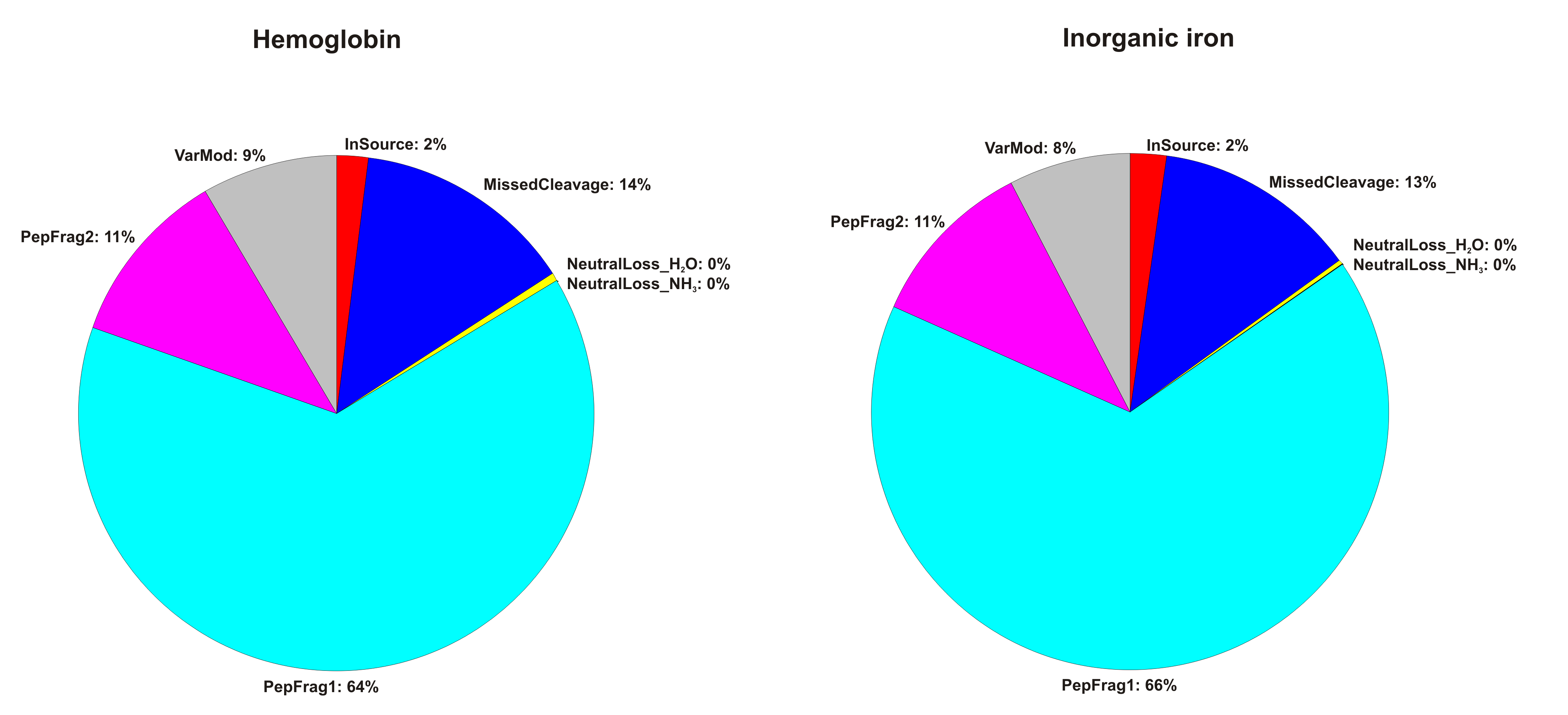

Supplement: Figure S3 — Peptide detection type that was used by the PLGS software. PLGS software uses an iterative search strategy for peptide identification as described previously [71]. During the first iteration (PepFrag1), only completely cleaved tryptic peptides are used for identification. The second pass of the database algorithm (PepFrag2) is designed to identify peptide modifications and nonspecific cleavage products to proteins that were positively identified in the first pass. VarMod: variable modifications. InSource: fragmentation that occurred on ionization source. MissedCleavage: missed cleavage performed by trypsin. NeutralLoss H2O and NH3 correspond to water and ammonia precursor losses. The Spotfire software was used to generate the graphics. (TIF) [file pntd.0002856.s003.tif]

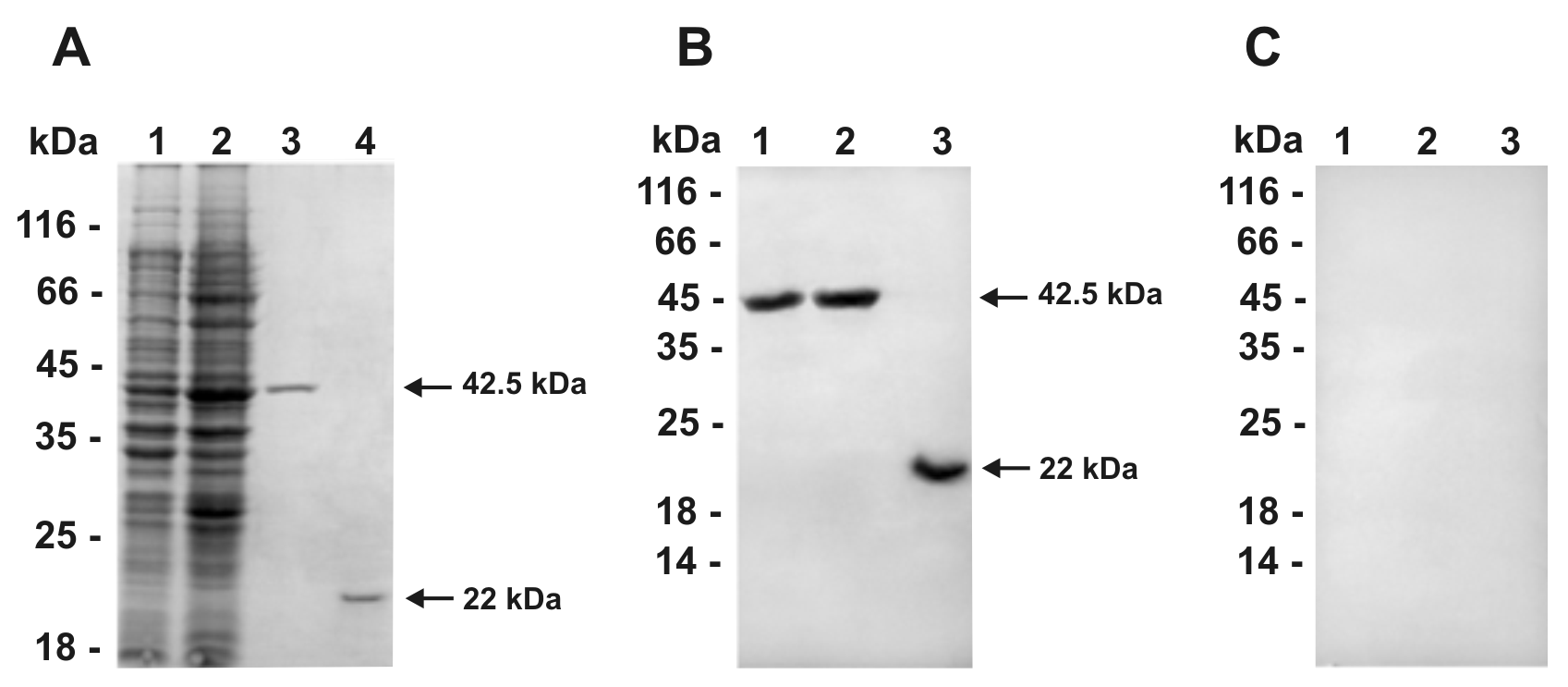

Supplement: Figure S4 — Expression and purification of the Pb 01 recombinant Rbt5 and the generation of a rabbit polyclonal antibody. A. SDS-PAGE analysis of Pb01 recombinant Rbt5. E. coli cells harboring the pGEX-4T-3-Rbt5 plasmid were grown to an OD600 of 0.8 and harvested before (lane 1) or after (lane 2) incubation with IPTG. The cells were lysed by sonication, the recombinant protein was purified (lane 3) and the fusion protein (glutathione S-transferase, GST) was cleaved by thrombin digestion (lane 4). Electrophoresis was performed on 10% SDS–PAGE, and the proteins were stained by Coomassie blue R-250. B and C. Western blot analysis of the recombinant Rbt5. The proteins that were obtained were screened using the rabbit polyclonal antibody anti-rRbt5 (B) or the rabbit preimmune serum (C). In B and C: E. coli C41 (DE3) that were transformed with the PGEX-4T-3-Rbt5 construct protein extract (lane 1); the affinity-isolated recombinant GST-Rbt5 (lane 2); the recombinant fusion protein cleaved with thrombin (lane 3). The reaction was developed using BCIP-NBT. Arrows indicate the deduced molecular mass of the proteins. Molecular markers are indicated at the left side of the panels. (TIF) [file pntd.0002856.s004.tif]

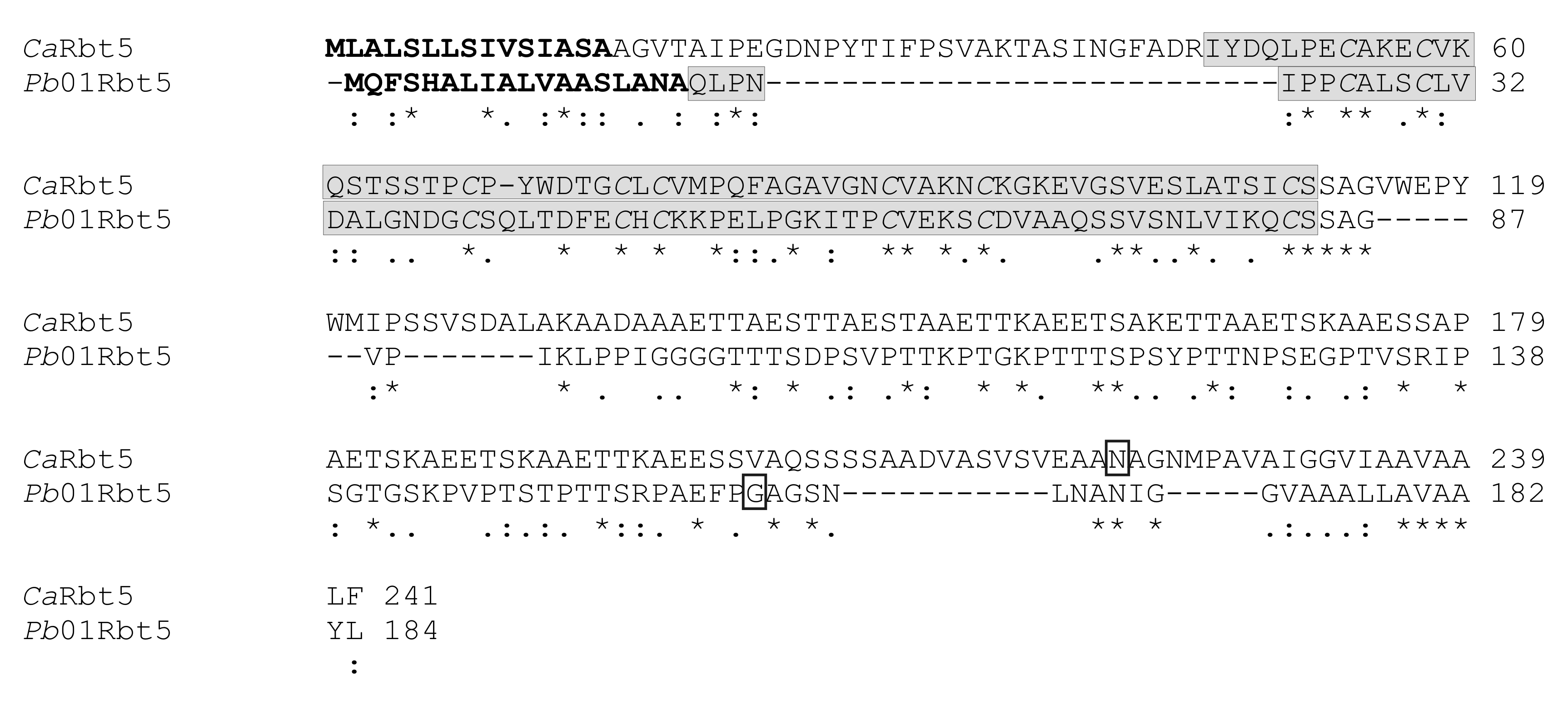

Supplement: Figure S5 — Pb 01 Rbt5 and Candida Rbt5 alignment. The amino acid sequences of the orthologs were aligned using the software ClustalX2. Asterisks: amino acid identity. Dots: conserved substitutions. In bold: signal peptide predicted by SignalP 4.1 Server. Grey box: CFEM domain that was predicted by the SMART online tool. In italic: cysteine residues inside the CFEM domain. Black border rectangle: omega-site that was predicted by the big-PI Fungal Predictor online tool. (TIF) [file pntd.0002856.s005.tif]

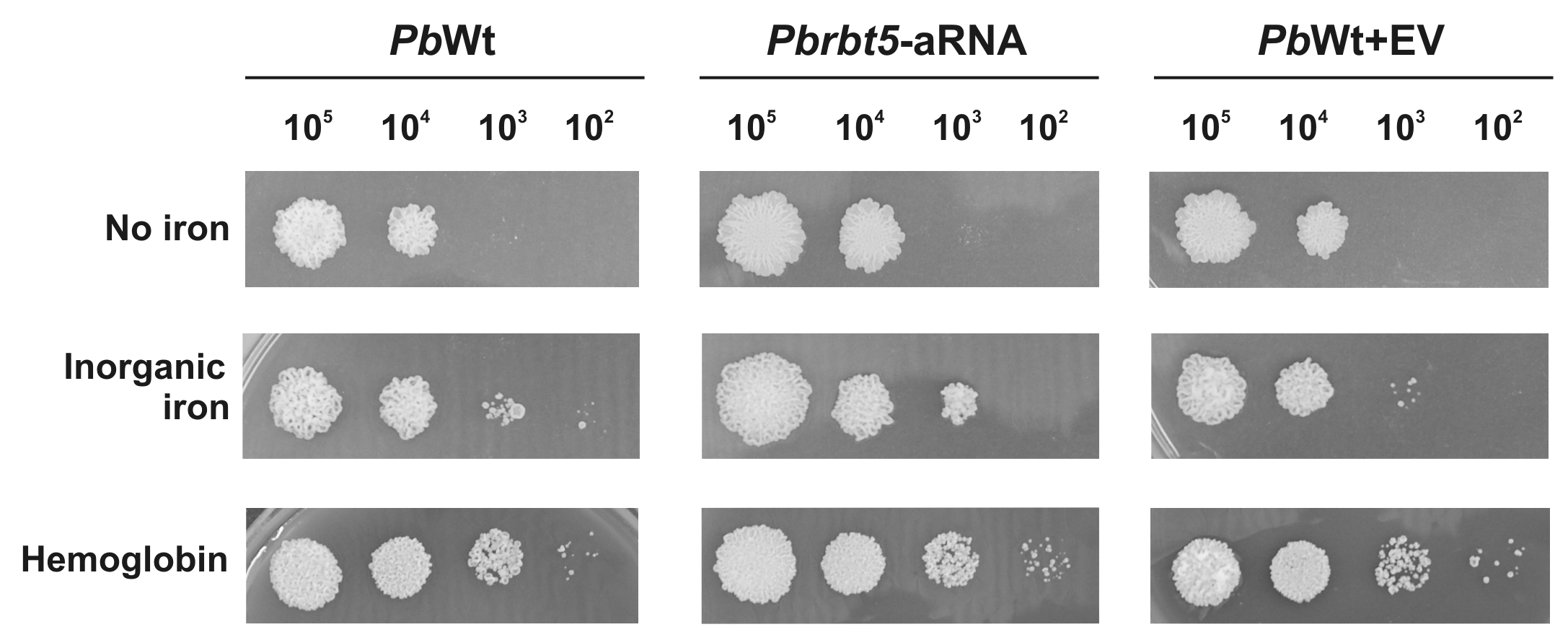

Supplement: Figure S6 — Paracoccidioides rbt5 knock down did not affect fungus growth under different iron availability conditions. Pb339 (PbWt), the rbt5 knock down strain (Pbrbt5-aRNA) and the Pb339 strain that was transformed with the pUR5750 empty vector (PbWt+EV) were collected after 36 h of iron scarcity, washed, and ten-fold serial dilutions of cell suspensions (105 to 102 cells) were spotted on MMcM medium plates that were supplemented with 50 µM BPS, which is an iron chelator. As indicated, 30 µM inorganic iron or 30 µM hemoglobin were added or not (no iron). (TIF) [file pntd.0002856.s006.tif]

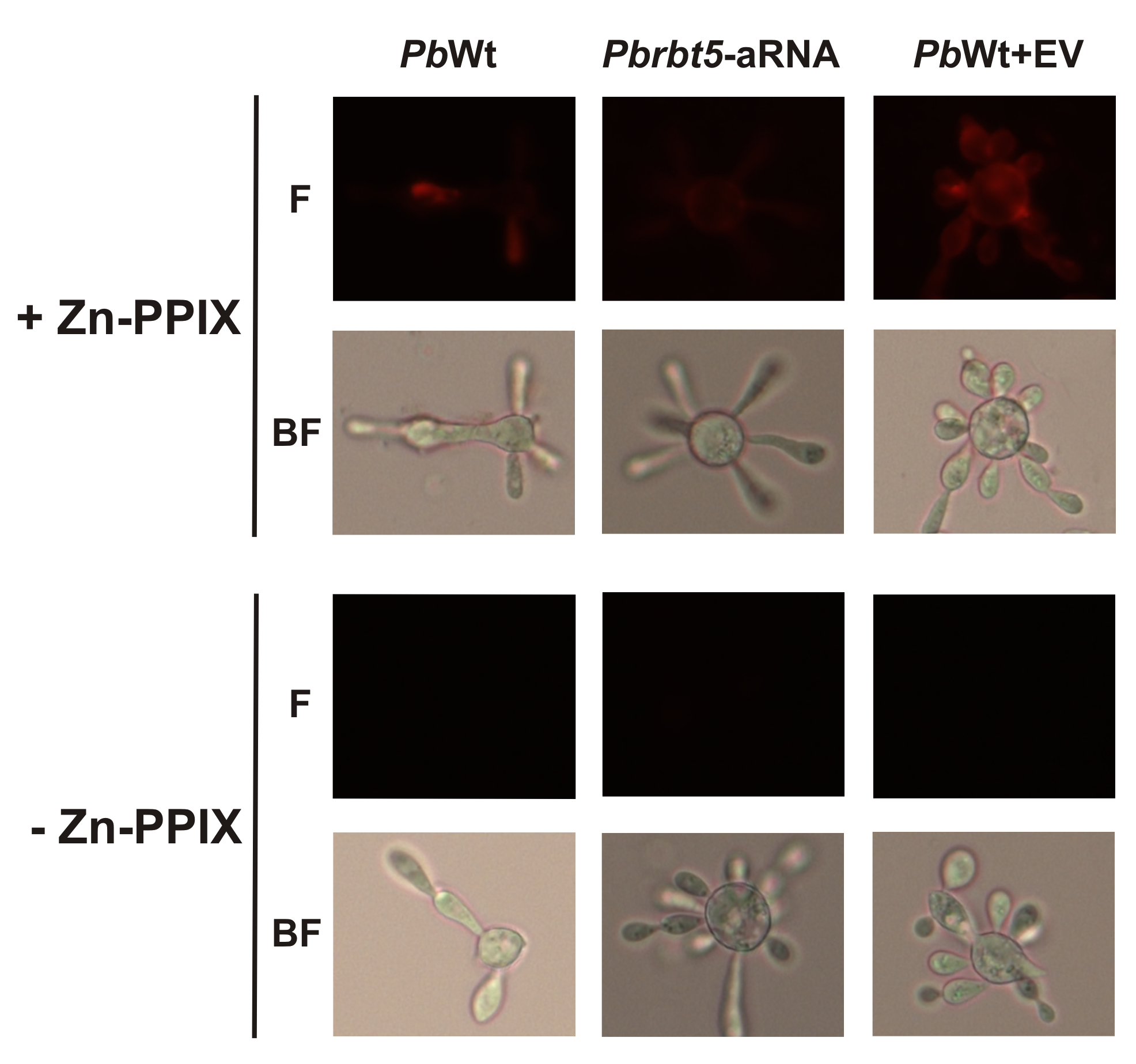

Supplement: Figure S7 — Paracoccidioides rbt5 knock down strain presents reduced Zn-PPIX uptake. Iron deprived Pb339 (PbWt), rbt5 knock down strain (Pbrbt5-aRNA) and Pb339 strain that was transformed with the pUR5750 empty vector (PbWt+EV) cells were incubated in MMcM medium supplemented (+) or not (−) with 60 µM zinc protoporphyrin IX (Zn-PPIX) for 2 h. After this period, the cells were washed twice, and observed by bright field microscopy (BF) and by live fluorescence microscopy (F). (TIF) [file pntd.0002856.s007.tif]
