# Supplementary material for: Hemoglobin Uptake by Paracoccidioides spp. Is Receptor-Mediated
Source: PLoS Negl Trop Dis. 2014 May 15;8(5):e2856. doi: 10.1371/journal.pntd.0002856 (PMC4022528; doi:10.1371/journal.pntd.0002856)
Supplement: Table S1 — Predicted members of the hemoglobin-receptor gene family in Paracoccidioides genus. (DOCX) [file pntd.0002856.s008.docx]

Supp. Table 1. Predicted members of the hemoglobin-receptor gene family in *Paracoccidioides* genus.

| **Orf**^a^ | **Gene name** | **Amino acids number**^a^ | **Signal peptide**^b^ | **Transmembranedomain**^c^ | **CFEM domain**^c^ | **GPI-anchored (predicted)**^d^ |
| --- | --- | --- | --- | --- | --- | --- |
| PAAG_05158 | Rbt5 | 184 | Yes | 0 | 1 | Yes |
| PAAG_02225 | Csa1/Wap1 | 453 | No | 0 | 1 | Yes |
| PAAG_01051 | Csa2 | 456 | Yes | 7 | 1 | No |
| PADG_05363 | Csa1/Wap1 | 289 | Yes | 0 | 1 | Yes |
| PABG_04599 | Rbt51 | 184 | Yes | 0 | 1 | Yes |
| PABG_01634 | Csa2 | 475 | No | 5 | 1 | No |

^a^Information obtained from *Paracoccidioides* Database (<http://www.broadinstitute.org/annotation/genome/paracoccidioides_brasiliensis/MultiHome.html>). Accession numbers: PAAG refers to *Pb*01, PADG refers to *Pb*18 and PABG refers to *Pb*03.

^b^*In silico* signal peptide prediction was performed with the online software SignalP 4.1 Server (<http://www.cbs.dtu.dk/services/SignalP/>).

^c^*In silico* protein analysis was performed with the online software SMART (<http://smart.embl-heidelberg.de/smart/set_mode.cgi?NORMAL=1>).

^d^GPI-anchor prediction was performed using the online software big-PI Fungal Predictor (<http://mendel.imp.ac.at/gpi/fungi_server.html>).
