# Supplementary material for: Hemoglobin Uptake by Paracoccidioides spp. Is Receptor-Mediated
Source: PLoS Negl Trop Dis. 2014 May 15;8(5):e2856. doi: 10.1371/journal.pntd.0002856 (PMC4022528; doi:10.1371/journal.pntd.0002856)
Supplement: Table S2 — Paracoccidioides Pb 01 proteins induced in presence of hemoglobin. (DOCX) [file pntd.0002856.s009.docx]

Supp. Table 2. *Paracoccidioides Pb*01 proteins induced in presence of hemoglobin.

|  | **Accession number^a^** | **Protein description** | **Score AVG** | **Peptides AVG** | **Fold change (Hb:Fe)** | **E.C. number** | **Subclassification** | **Filter^b^** |
| --- | --- | --- | --- | --- | --- | --- | --- | --- |
| **METABOLISM** | | | | | | | | |
| **Amino acid metabolism** | | | | | | | | |
|  | PAAG_02163 | acetyl-/propionyl-coenzyme A carboxylase alpha chain | 249.65 | 16.33 | *** | 6.4.1.3 | Valine and isoleucine degradation | 1 |
|  | PAAG_07036 | methylmalonate-semialdehyde dehydrogenase | 279.14 | 14.83 | 1.60 | 1.2.1.27 | Valine, leucine and isoleucine degradation | 2 |
|  | PAAG_00221 | acetolactate synthase | 260.71 | 10.20 | 1.55 | 2.2.1.6 | Valine, leucine and isoleucine biosynthesis | 2 |
|  | PAAG_06416 | conserved hypothetical protein (alanine racemase) | 169.27 | 4.00 | *** | 5.1.1.1 | Alanine metabolism | 1 |
|  | PAAG_08065 | aspartate-semialdehyde dehydrogenase | 501.59 | 5.80 | 3.35 | 1.2.1.11 | Amino acid biosynthesis | 2 |
|  | PAAG_03138 | alanine-glyoxylate aminotransferase | 381.01 | 7.33 | 1.40 | 2.6.1.44 | Amino acid metabolism | 2 |
|  | PAAG_06217 | acetylornithine aminotransferase | 313.28 | 11.25 | 1.72 | 2.6.1.11 | Arginine biosynthesis | 1 |
|  | PAAG_06506 | aspartate aminotransferase | 253.11 | 6.50 | 1.42 | 2.6.1.1 | Aspartate and glutamate metabolism | 1 |
|  | PAAG_06835 | cystathionine gamma-lyase | 257.79 | 7.00 | *** | 4.4.1.1 | Cysteine biosynthesis | 1 |
|  | PAAG_07813 | cysteine synthase | 310.64 | 7.33 | *** | 4.2.1.22 | Cysteine biosynthesis | 2 |
|  | PAAG_05392 | betaine aldehyde dehydrogenase | 913.69 | 8.67 | 1.68 | 1.2.1.8 | Glicine biosynthesis | 2 |
|  | PAAG_01568 | glycine dehydrogenase | 193.28 | 16.00 | *** | 1.4.4.2 | Glycine degradation | 2 |
|  | PAAG_05406 | histidine biosynthesis trifunctional protein | 196.65 | 14.00 | *** | 3.5.4.19; 3.6.1.31; 1.1.1.23 | Histidine biosynthesis | 1 |
|  | PAAG_00285 | imidazole glycerol phosphate synthase hisHF | 175.08 | 19.00 | *** | 2.4.2.-; 4.1.3.- | Histidine biosynthesis | 1 |
|  | PAAG_09095 | ATP phosphoribosyltransferase | 1303.26 | 4.67 | 1.79 | 2.4.2.17 | Histidine biosynthesis | 2 |
|  | PAAG_04099 | methylcrotonoyl-CoA carboxylase subunit alpha | 179.63 | 10.00 | *** | 6.4.1.4 | Leucine degradation | 1 |
|  | PAAG_06387 | homoisocitrate dehydrogenase | 431.93 | 8.00 | 1.84 | 1.1.1.87 | Lysine biosynthesis | 2 |
|  | PAAG_02693 | saccharopine dehydrogenase | 241.77 | 12.67 | 1.43 | 1.5.1.10 | Lysine metabolism | 1 |
|  | PAAG_07626 | Cobalamin-independent synthase | 1296.09 | 25.33 | 2.92 | 2.1.1.14 | Methionine biosynthesis | 2 |
|  | PAAG_06996 | G-protein comlpex beta subunit CpcB | 1162.11 | 11.33 | 2.32 | N.A. | Regulation of amino acid metabolism | 2 |
|  | PAAG_03613 | phosphoserine aminotransferase | 265.03 | 8.67 | 4.35 | 2.6.1.52 | Serine biosynthesis | 2 |
|  | PAAG_07760 | threonine synthase | 171.92 | 8.00 | *** | 4.2.3.1 | Threonine biosynthesis | 1 |
|  | PAAG_08668 | anthranilate synthase component 2 | 242.62 | 11.50 | *** | 4.1.3.27 | Tryptophan biosynthesis | 1 |
|  | PAAG_05005 | anthranilate synthase component 1 | 186.39 | 14.00 | 1.38 | 4.1.3.27 | Tryptophan biosynthesis | 2 |
|  | PAAG_02644 | kynurenine-oxoglutarate transaminase | 179.07 | 6.00 | *** | 2.6.1.7 | Tryptophan degradation | 2 |
|  | PAAG_08164 | homogentisate 1,2-dioxygenase | 323.07 | 8.25 | 1.68 | 1.13.11.5 | Tyrosine degradation | 1 |
| **Nitrogen, sulfur and selenium metabolism** | | | | | | | | |
|  | PAAG_00468 | 4-aminobutyrate aminotransferase | 1947.94 | 10.50 | 1.86 | 2.6.1.19 | Nitrogen utilization | 2 |
|  | PAAG_03333 | formamidase | 14545.80 | 19.33 | 1.22 | 3.5.1.49 | Nitrogen compound metabolic process | 2 |
|  | PAAG_05929 | sulfate adenylyltransferase | 303.58 | 9.20 | 2.39 | 2.7.7.4 | Sulfur metabolism | 2 |
| **Nucleotide/nucleoside/nucleobase metabolism** | | | | | | | | |
|  | PAAG_05018 | allantoate amidohydrolase | 261.39 | 8.00 | *** | 3.5.3.9 | Purine metabolism | 2 |
|  | PAAG_01751 | conserved hypothetical protein (cytidine deaminase) | 423.39 | 7.00 | *** | 3.5.4.5 | Pyrimidine metabolism | 2 |
| **C-compound and carbohydrate metabolism** | | | | | | | | |
|  | PAAG_00050 | pyruvate dehydrogenase protein X component | 1118.49 | 11.50 | 1.93 | N.A. | Acetyl-CoA biosynthesis | 2 |
|  | PAAG_03330 | dihydrolipoyl dehydrogenase | 2921.00 | 20.33 | 1.26 | 1.8.1.4 | Acetyl-CoA biosynthesis | 2 |
|  | PAAG_01931 | phosphoacetylglucosamine mutase | 185.26 | 6.00 | *** | 5.4.2.3 | Chitin biosynthetic process | 2 |
|  | PAAG_06057 | conserved hypothetical protein (aldose 1-epimerase) | 280.05 | 4.00 | *** | 5.1.3.3 | Hexose metabolism | 2 |
|  | PAAG_03243 | conserved hypothetical protein (aldose 1-epimerase) | 627.33 | 6.83 | 1.28 | 5.1.3.3 | Hexose metabolism | 2 |
|  | PAAG_01347 | actin cytoskeleton protein (VIP1) | 390.56 | 10.00 | 2.18 | 2.7.4.21; 2.7.4.24 | Inositol phosphate biosynthesis | 1 |
|  | PAAG_04550 | 2-methylcitrate synthase | 5518.20 | 20.17 | 4.53 | 2.3.3.5 | Methylcitrate cycle | 2 |
|  | PAAG_04290 | neutral alpha-glucosidase AB | 218.88 | 13.33 | 1.35 | 3.2.1.20 | Polysaccharide metabolism | 1 |
|  | PAAG_00435 | (R)-benzylsuccinyl-CoA dehydrogenase | 274.05 | 10.50 | 2.23 | 1.3.8.3 | Toluene degradation | 1 |
|  | PAAG_06817 | UTP-glucose-1-phosphate uridylyltransferase | 445.48 | 14.67 | *** | 2.7.7.9 | UDP-glucose metabolic process | 2 |
|  | PAAG_02011 | phosphoglucomutase | 505.72 | 23.17 | 1.52 | 5.4.2.2 | UDP-glucose metabolic process | 2 |
| **Lipid, fatty acid and isoprenoid metabolism** | | | | | | | | |
|  | PAAG_07786 | acetyl-CoA acetyltransferase | 1409.07 | 7.83 | 1.73 | 2.3.1.9 | Ergosterol biosynthesis | 2 |
|  | PAAG_03689 | 3-ketoacyl-CoA thiolase B | 232.09 | 9.67 | *** | 2.3.1.16 | Fatty acid beta-oxidation | 1 |
|  | PAAG_05690 | esterase D | 227.18 | 7.00 | *** | 3.1.1.1 | Fatty acid biosynthesis | 1 |
|  | PAAG_07631 | short chain dehydrogenase/reductase family protein | 240.97 | 8.00 | *** | 1.1.1.100 | Fatty acid biosynthesis | 2 |
|  | PAAG_04811 | 2-hydroxyacyl-CoA lyase | 236.87 | 10.50 | *** | 4.1.-.- | Fatty acid metabolism | 2 |
|  | PAAG_06953 | short chain dehydrogenase/reductase family | 303.51 | 5.00 | 1.22 | 1.1.1.300 | Glycolipid metabolism | 2 |
|  | PAAG_03960 | isopentenyl-diphosphate Delta-isomerase | 196.53 | 8.00 | *** | 5.3.3.2 | Isoprenoid metabolism | 1 |
|  | PAAG_06215 | hydroxymethylglutaryl-CoA lyase | 735.72 | 7.17 | 1.35 | 4.1.3.4 | Lipid metabolic process | 2 |
| **Metabolism of vitamins, cofactors and prosthetic groups** | | | | | | | | |
|  | PAAG_02352 | molybdopterin binding domain-containing protein | 167.28 | 6.00 | *** | N.A. | Molybdenum cofactor biosynthesis | 1 |
|  | PAAG_08856 | nicotinate-nucleotide pyrophosphorylase | 147.96 | 2.00 | *** | 2.4.2.19 | De novo NAD biosynthesis | 1 |
| **Secondary metabolism** | | | | | | | | |
|  | PAAG_01244 | beta-lactamase family protein | 511.77 | 7.67 | 2.16 | 3.5.2.6 | Beta-lactam degradation | 2 |
|  | PAAG_04478 | dienelactone hydrolase family protein | 512.91 | 7.20 | 1.77 | 3.1.1.45 | Secondary metabolites biosynthesis | 2 |
|  | PAAG_04443 | spermidine synthase | 410.25 | 6.33 | 1.27 | 2.5.1.16 | Amines metabolism | 2 |
| **ENERGY** | | | | | | | | |
| **Glycolysis and gluconeogenesis** | | | | | | | | |
|  | PAAG_08203 | phosphoenolpyruvate carboxykinase | 163.90 | 8.00 | *** | 4.1.1.49 | Gluconeogenesis | 1 |
|  | PAAG_06380 | pyruvate kinase | 1155.19 | 18.33 | 2.14 | 2.7.1.40 | Glycolysis | 2 |
|  | PAAG_00771 | enolase | 41257.25 | 25.67 | *** | 4.2.1.11 | Glycolysis/ Gluconeogenesis | 2 |
| **Pentose-phosphate pathway** | | | | | | | | |
|  | PAAG_01178 | 6-phosphogluconate dehydrogenase | 1134.90 | 14.33 | 1.70 | 1.1.1.44 | Oxidative branch | 2 |
|  | PAAG_04444 | transketolase | 1685.75 | 23.67 | 1.40 | 2.2.1.1 | Non-oxidative branch | 2 |
| **Tricarboxylic-acid cycle** | | | | | | | | |
|  | PAAG_07729 | isocitrate dehydrogenase subunit 2 | 184.77 | 6.00 | *** | 1.1.1.41 | TCA cycle | 1 |
|  | PAAG_01725 | succinate dehydrogenase flavoprotein subunit | 223.67 | 8.50 | *** | 1.3.5.1 | TCA cycle | 2 |
|  | PAAG_02732 | 2-oxoglutarate dehydrogenase E1 | 276.45 | 18.25 | 1.62 | 1.2.4.2 | TCA cycle | 1 |
|  | PAAG_08915 | dihydrolipoamide succinyltransferase | 749.88 | 13.33 | 1.27 | 2.3.1.61 | TCA cycle | 2 |
|  | PAAG_00417 | succinyl-CoA ligase subunit alpha | 430.44 | 6.33 | 1.26 | 6.2.1.5 | TCA cycle | 2 |
| **Electron transport and membrane-associated energy conservation** | | | | | | | | |
|  | PAAG_02297 | cytochrome b-c1 complex subunit Rieske | 155.75 | 3.00 | *** | 1.10.2.2 | Electron transport chain | 1 |
|  | PAAG_04931 | electron transfer flavoprotein subunit beta | 389.96 | 7.00 | *** | N.A. | Electron transport chain | 2 |
|  | PAAG_08088 | cytochrome b-c1 complex subunit 2 | 280.22 | 7.00 | *** | N.A. | Electron transport chain | 2 |
|  | PAAG_02382 | quinone oxidoreductase | 307.88 | 8.00 | 1.73 | 1.6.5.3 | Electron transport chain | 1 |
|  | PAAG_06796 | conserved hypothetical protein (cytochrome c oxidase subunit) | 1238.58 | 6.50 | 1.20 | 1.9.3.1 | Electron transport chain | 2 |
| **Respiration** | | | | | | | | |
|  | PAAG_03309 | suaprga1 | 1475.52 | 8.33 | 1.30 | N.A. | Aerobic respiration | 2 |
| **Fermentation** | | | | | | | | |
|  | PAAG_02050 | pyruvate decarboxylase | 2099.43 | 16.17 | 2.75 | 4.1.1.1 | Alcoholic fermentation | 2 |
|  | PAAG_05249 | aldehyde dehydrogenase | 5638.29 | 23.50 | 2.16 | 1.2.1.3 | Alcoholic fermentation | 2 |
| **Energy conservation and regeneration** | | | | | | | | |
|  | PAAG_08082 | plasma membrane ATPase | 207.96 | 5.00 | *** | 3.6.3.6 | ATP biosynthesis | 2 |
|  | PAAG_05576 | ATP synthase gamma chain | 1480.68 | 7.80 | 2.03 | 3.6.3.14 | ATP biosynthesis | 2 |
|  | PAAG_04820 | ATPase alpha subunit | 5155.57 | 21.50 | 1.92 | 3.6.3.14 | ATP biosynthesis | 2 |
|  | PAAG_08037 | ATP synthase subunit beta | 8785.14 | 29.33 | 1.22 | 3.6.3.14 | ATP biosynthesis | 2 |
| **CELL RESCUE, DEFENSE AND VIRULENCE** | | | | | | | | |
| **Stress response** | | | | | | | | |
|  | PAAG_02116 | Hsp70 | 385.88 | 16.50 | 1.35 | N.A. | Heat shock response | 2 |
|  | PAAG_08152 | 3',5'-bisphosphate nucleotidase | 155.76 | 7.00 | *** | 3.1.3.7 | Hyperosmotic salinity response | 1 |
|  | PAAG_06947 | gamma-glutamyltranspeptidase | 204.85 | 12.00 | *** | 2.3.2.2 | Nitrogen starvation response | 2 |
|  | PAAG_00997 | actin-interacting protein | 178.28 | 12.00 | *** | N.A. | Osmotic stress response | 1 |
|  | PAAG_07020 | thioredoxin reductase | 340.22 | 7.00 | *** | 1.8.1.9 | Oxidative stress response | 2 |
|  | PAAG_05061 | AhpC/TSA family protein | 763.03 | 4.67 | *** | N.A. | Oxidative stress response | 2 |
|  | PAAG_09083 | TCTP family protein | 3094.32 | 6.83 | 1.72 | N.A. | Oxidative stress response | 2 |
|  | PAAG_03931 | glutathione S-transferase Gst3 | 1064.17 | 13.00 | 1.49 | 2.5.1.18 | Oxidative stress response | 2 |
| **CELL CYCLE AND DNA PROCESSING** | | | | | | | | |
| **DNA processing** | | | | | | | | |
|  | PAAG_04389 | mating-type switching protein swi10 | 224.32 | 6.00 | *** | N.A. | DNA repair | 1 |
|  | PAAG_07099 | histone H3.3 | 1397.91 | 3.00 | *** | N.A. | DNA repair | 2 |
|  | PAAG_01596 | chromatin remodeling complex subunit (Arp5) | 176.87 | 13.00 | *** | N.A. | DNA repair | 1 |
|  | PAAG_00773 | DNA damage checkpoint protein rad24 | 1991.61 | 9.83 | 3.90 | N.A. | DNA repair | 2 |
|  | PAAG_06751 | DNA damage checkpoint protein rad24 | 3506.98 | 13.00 | 2.51 | N.A. | DNA repair | 2 |
|  | PAAG_00923 | proliferating cell nuclear antigen | 1264.29 | 9.00 | 1.60 | N.A. | DNA synthesis and replication | 2 |
|  | PAAG_07098 | histone H4.1 | 1662.13 | 5.00 | *** | N.A. | Nucleosome assembly | 2 |
|  | PAAG_00126 | histone H4.2 | 1685.65 | 5.33 | *** | N.A. | Nucleosome assembly | 2 |
| **Cell cycle** | | | | | | | | |
|  | PAAG_05518 | cell division cycle protein | 368.50 | 18.00 | *** | N.A. | Cell cycle | 2 |
| **TRANSCRIPTION** | | | | | | | | |
| **RNA synthesis** | | | | | | | | |
|  | PAAG_08471 | histone H2A.Z | 184.61 | 4.00 | *** | N.A. | Transcription regulation | 2 |
|  | PAAG_01710 | polymerase II polypeptide D | 245.71 | 4.00 | *** | 2.7.7.6 | Transcription | 1 |
| **PROTEIN SYNTHESIS** | | | | | | | | |
| **Ribosome biogenesis** | | | | | | | | |
|  | PAAG_01433 | 40S ribosomal protein S14 | 707.87 | 5.33 | 1.25 | N.A. | Translation | 2 |
|  | PAAG_09043 | 40S ribosomal protein S2 | 1159.36 | 7.83 | 1.60 | N.A. | Translation | 2 |
|  | PAAG_07847 | 40S ribosomal protein S26 | 1787.39 | 1.60 | 1.35 | N.A. | Translation | 2 |
|  | PAAG_03828 | 40S ribosomal protein S9 | 1081.01 | 7.00 | 1.27 | N.A. | Translation | 2 |
|  | PAAG_01052 | 60S ribosomal protein L10-B | 671.85 | 5.80 | 1.43 | N.A. | Translation | 2 |
|  | PAAG_06320 | 60S ribosomal protein L13 | 3696.57 | 8.50 | 1.26 | N.A. | Translation | 2 |
|  | PAAG_00969 | 60S ribosomal protein L15 | 3234.57 | 6.33 | 1.28 | N.A. | Translation | 2 |
|  | PAAG_01834 | 60S ribosomal protein L16 | 912.15 | 8.50 | 1.22 | N.A. | Translation | 2 |
|  | PAAG_05379 | 60S ribosomal protein L17 | 2959.17 | 5.33 | 1.38 | N.A. | Translation | 2 |
|  | PAAG_00430 | 60S ribosomal protein L2 | 2042.63 | 7.17 | 1.36 | N.A. | Translation | 2 |
|  | PAAG_07385 | 60S ribosomal protein L23a | 1911.74 | 4.67 | 1.20 | N.A. | Translation | 2 |
|  | PAAG_00765 | 60S ribosomal protein L36 | 2645.63 | 6.33 | 1.28 | N.A. | Translation | 2 |
|  | PAAG_07550 | 60S ribosomal protein L44 | 1230.65 | 3.00 | *** | N.A. | Translation | 2 |
|  | PAAG_06487 | 60S ribosomal protein L7-C | 2051.46 | 9.50 | 1.25 | N.A. | Translation | 2 |
|  | PAAG_04998 | 60S ribosomal protein L8-B | 2603.35 | 12.83 | 1.28 | N.A. | Translation | 2 |
|  | PAAG_00347 | 60S ribosomal protein L9-B | 476.25 | 5.00 | *** | N.A. | Translation | 2 |
|  | PAAG_06743 | ribosomal protein L23e | 795.79 | 5.00 | *** | N.A. | Translation | 2 |
|  | PAAG_05051 | conserved hypothetical protein (ribosomal protein S17) | 188.74 | 6.00 | *** | N.A. | Translation | 1 |
|  | PAAG_01001 | hypothetical protein (40S ribosomal protein S29) | 217.83 | 3.67 | 1.28 | N.A. | Translation | 2 |
| **Translation** | | | | | | | | |
|  | PAAG_02921 | elongation factor Tu | 234.53 | 13.00 | *** | N.A. | Translation | 2 |
|  | PAAG_06084 | eukaryotic translation initiation factor 3 subunit B | 162.02 | 10.00 | *** | N.A. | Translation initiation | 1 |
|  | PAAG_03556 | elongation factor 1-gamma 1 | 223.88 | 8.00 | *** | N.A. | Translation elongation | 1 |
|  | PAAG_00594 | elongation factor 2 | 187.27 | 15.33 | *** | N.A. | Translation elongation | 1 |
|  | PAAG_01665 | PKHD-type hydroxylase TPA1 | 156.35 | 7.00 | *** | 1.14.11.- | Translation termination | 1 |
| **PROTEIN FATE (folding, modification, destination)** | | | | | | | | |
| **Protein folding and stabilization** | | | | | | | | |
|  | PAAG_06168 | peptidyl-prolyl cis-trans isomerase cypE | 805.88 | 5.17 | 1.36 | 5.2.1.8 | Protein folding | 2 |
|  | PAAG_06255 | mitochondrial co-chaperone GrpE | 1865.43 | 8.20 | 1.35 | N.A. | Protein folding | 2 |
|  | PAAG_05226 | Hsp90 binding co-chaperone (Sba1) | 3452.01 | 7.67 | 1.26 | N.A. | Protein folding | 2 |
|  | PAAG_07409 | conserved hypothetical protein (prefoldin subunit 6) | 330.41 | 4.00 | *** | N.A. | Protein folding | 2 |
| **Protein targeting, sorting and translocation** | | | | | | | | |
|  | PAAG_01854 | hypothetical protein (nuclear transport factor 2) | 621.37 | 1.50 | *** | N.A. | Protein import into nucleus | 2 |
| **Protein modification** | | | | | | | | |
|  | PAAG_04195 | ubiquitin-conjugating enzyme | 250.71 | 1.00 | *** | N.A. | Ubiquitination | 1 |
|  |  |  |  |  |  |  |  |  |
| **Protein/peptide degradation** | | | | | | | | |
|  | PAAG_02907 | conserved hypothetical protein (ankyrin repeat protein) | 288.17 | 6.00 | *** | N.A. | Cytoplasmic and nuclear protein degradation | 1 |
|  | PAAG_03512 | carboxypeptidase Y | 496.47 | 6.33 | 1.23 | 3.4.16.5 | Lysossomal and vacuolar protein degradation | 2 |
|  | PAAG_01966 | hypothetical protein (vacuolar protease A) | 299.75 | 3.33 | 1.25 | 3.4.23.25 | Lysossomal and vacuolar protein degradation | 2 |
| **PROTEIN WITH BINDING FUNCTION OR COFACTOR REQUIREMENT** | | | | | | | | |
| **Nucleic acid binding** | | | | | | | | |
|  | PAAG_05905 | conserved hypothetical protein (dsDNA-binding protein PDCD5) | 240.98 | 3.00 | *** | N.A. | DNA binding | 1 |
|  | PAAG_04913 | conserved hypothetical protein (RNP domain-containing protein) | 2396.12 | 9.83 | 1.43 | N.A. | DNA binding | 2 |
| **Structural protein binding** | | | | | | | | |
|  | PAAG_00004 | actin binding protein | 570.45 | 12.83 | 2.01 | N.A. | Actin filament binding | 2 |
|  | PAAG_01764 | actin binding protein | 288.22 | 9.00 | 1.58 | N.A. | Actin filament binding | 1 |
| **Metal binding** | | | | | | | | |
|  | PAAG_03314 | RING finger domain-containing protein | 157.32 | 4.00 | *** | N.A. | Zinc binding | 1 |
|  | PAAG_00976 | LiPid Depleted family member | 195.69 | 5.00 | *** | N.A. | Iron binding | 1 |
| **Nucleotide/nucleoside/nucleobase binding** | | | | | | | | |
|  | PAAG_05623 | conserved hypothetical protein (protein kinase) | 208.36 | 9.00 | *** | N.A. | ATP binding | 2 |
| **CELLULAR TRANSPORT, TRANSPORT FACILITIES AND TRANSPORT ROUTES** | | | | | | | | |
| **Transported compounds** | | | | | | | | |
|  | PAAG_04276 | phosphatidylinositol transporter | 1310.76 | 10.83 | 1.65 | N.A. | Golgi protein transport | 2 |
|  | PAAG_01051 | conserved hypothetical protein (*Pb*01 Csa2) | 151.83 | 6.00 | *** | N.A. | Hemoglobin receptor | 1 |
| **INTERACTION WITH THE ENVIRONMENT** | | | | | | | | |
| **Homeostasis** | | | | | | | | |
|  | PAAG_05851 | cysteine desulfurase | 160.27 | 7.00 | *** | 2.8.1.7 | Iron-sulfur cluster assembly | 1 |
|  | PAAG_05850 | conserved hypothetical protein (cysteine desulfurase) | 395.45 | 10.25 | 1.65 | 2.8.1.7 | Iron-sulfur cluster assembly | 1 |
| **BIOGENESIS OF CELLULAR COMPONENTS** | | | | | | | | |
| **Cell wall** | | | | | | | | |
|  | PAAG_07670 | cell wall protein ECM33 precursor | 347.01 | 6.67 | *** | N.A. | Cell wall organization | 2 |
|  | PAAG_05068 | 1,3-beta-glucanosyltransferase gel4 | 159.84 | 10.00 | *** | 2.4.1.- | Cell wall organization | 1 |
|  | PAAG_05763 | conserved hypothetical protein (acid phosphatase) | 456.10 | 7.50 | 1.92 | 3.1.3.2 | Anchored to membrane | 2 |
| **UNCLASSIFIED PROTEINS** | | | | | | | | |
|  | PAAG_08235 | predicted protein | 393.54 | 5.00 | *** | N.A. | N.A. | 1 |
|  |  |  |  |  |  |  |  |  |
|  | PAAG_07397 | conserved hypothetical protein | 185.29 | 5.00 | *** | N.A. | N.A. | 1 |
|  | PAAG_07322 | predicted protein | 268.59 | 2.00 | *** | N.A. | N.A. | 1 |
|  | PAAG_06963 | hypothetical protein | 244.60 | 1.00 | *** | N.A. | N.A. | 1 |
|  | PAAG_05977 | predicted protein | 216.15 | 4.00 | *** | N.A. | N.A. | 2 |
|  | PAAG_05578 | predicted protein | 451.29 | 6.00 | *** | N.A. | N.A. | 2 |
|  | PAAG_02285 | predicted protein | 195.50 | 4.00 | *** | N.A. | N.A. | 1 |
|  | PAAG_01245 | predicted protein | 306.96 | 2.00 | *** | N.A. | N.A. | 1 |
|  | PAAG_00798 | predicted protein | 257.69 | 6.00 | *** | N.A. | N.A. | 1 |
|  | PAAG_00324 | conserved hypothetical protein | 241.69 | 7.00 | *** | N.A. | N.A. | 1 |
|  | PAAG_00250 | conserved hypothetical protein | 357.98 | 1.00 | *** | N.A. | N.A. | 1 |
|  | PAAG_00090 | conserved hypothetical protein | 335.29 | 6.00 | *** | N.A. | N.A. | 2 |
|  | PAAG_01045 | conserved hypothetical protein | 375.12 | 3.67 | 4.01 | N.A. | N.A. | 2 |
|  | PAAG_03066 | conserved hypothetical protein (putative cyclase) | 982.37 | 7.83 | 1.88 | N.A. | N.A. | 2 |
|  | PAAG_03701 | BAR domain-containing protein | 628.35 | 9.00 | 1.60 | N.A. | N.A. | 2 |
|  | PAAG_07875 | conserved hypothetical protein | 11074.60 | 8.17 | 1.20 | N.A. | N.A. | 2 |

^a^Information obtained from *Paracoccidioides* Database (<http://www.broadinstitute.org/annotation/genome/paracoccidioides_brasiliensis/MultiHome.html>)

^b^filter 1 – proteins derived from PepFrag2; filter 2 – proteins derived from PepFrag1, as determined by PLGS and cited by Murad and Rech (2012).

***: proteins identified just in presence of hemoglobin;

N.A.: not applicable.
