# Supplementary material for: Hemoglobin Uptake by Paracoccidioides spp. Is Receptor-Mediated
Source: PLoS Negl Trop Dis. 2014 May 15;8(5):e2856. doi: 10.1371/journal.pntd.0002856 (PMC4022528; doi:10.1371/journal.pntd.0002856)
Supplement: Table S3 — Paracoccidioides Pb 01 proteins repressed in presence of hemoglobin. (DOCX) [file pntd.0002856.s010.docx]

Supp. Table 3. *Paracoccidioides Pb*01 proteins repressed in presence of hemoglobin.

|  | **Accession number** | **Protein description** | **Score AVG** | **Peptides AVG** | **Fold change (Hb:Fe)** | **E.C. number** | **Subclassification** | **Filter** |
| --- | --- | --- | --- | --- | --- | --- | --- | --- |
| **METABOLISM** | | | | | | | | |
| **Amino acid metabolism** | | | | | | | | |
|  | PAAG_01206 | L-asparaginase | 197.88 | 5.00 | *** | 3.5.1.1 | Asparagine degradation | 1 |
|  | PAAG_01365 | choline dehydrogenase | 206.25 | 13.00 | *** | 1.1.99.1 | Glycine biosynthesis | 1 |
|  | PAAG_02935 | glycine cleavage system H protein | 404.32 | 4.33 | 0.66 | N.A. | Glycine degradation | 2 |
|  | PAAG_04102 | isovaleryl-CoA dehydrogenase | 330.74 | 7.00 | *** | 1.3.8.4 | Leucine degradation | 2 |
|  | PAAG_01974 | mitochondrial methylglutaconyl-CoA hydratase | 223.92 | 6.00 | *** | 4.2.1.18 | Leucine degradation | 1 |
|  | PAAG_03569 | 1,2-dihydroxy-3-keto-5-methylthiopentene dioxygenase | 175.60 | 6.00 | *** | 1.13.11.54 | Methionine biosynthesis | 1 |
|  | PAAG_08166 | 4-hydroxyphenylpyruvate dioxygenase | 203.44 | 3.00 | *** | 1.13.11.27 | Tyrosine and phenylalanine degradation | 1 |
|  | PAAG_00014 | dihydroxy-acid dehydratase | 312.52 | 10.83 | 0.73 | 4.2.1.9 | Valine and isoleucine biosynthesis | 2 |
|  | PAAG_02554 | 3-hydroxyisobutyryl-CoA hydrolase | 402.87 | 9.75 | 0.58 | 3.1.2.4 | Valine degradation | 2 |
|  | PAAG_01194 | 2-oxoisovalerate dehydrogenase subunit beta | 219.86 | 6.00 | *** | 1.2.4.4 | Valine, leucine and isoleucine degradation | 2 |
|  | PAAG_06096 | phospho-2-dehydro-3-deoxyheptonate aldolase | 1370.44 | 9.67 | 0.66 | 2.5.1.54 | Phenyalanine, tyrosine and tryptophan biosynthesis | 2 |
|  | PAAG_07659 | chorismate synthase | 193.84 | 3.50 | 0.61 | 4.2.3.5 | Phenyalanine, tyrosine and tryptophan biosynthesis | 2 |
| **Nitrogen, sulfur and selenium metabolism** | | | | | | | | |
|  | PAAG_04525 | glutamine synthetase | 156.10 | 9.00 | *** | 6.3.1.2 | Nitrogen metabolism | 1 |
|  | PAAG_07689 | NADP-specific glutamate dehydrogenase | 2006.41 | 19.83 | 0.75 | 1.4.1.4 | Nitrogen metabolism | 2 |
| **Nucleotide/nucleoside/nucleobase metabolism** | | | | | | | | |
|  | PAAG_04297 | thymidylate kinase | 359.06 | 5.00 | *** | 2.7.4.9 | Pyrimidine biosynthesis | 2 |
|  | PAAG_02336 | conserved hypothetical protein (NUDIX hydrolase) | 238.53 | 8.00 | *** | 3.6.1.- | Nucleoside diphosphate metabolism | 1 |
| **C-compound and carbohydrate metabolism** | | | | | | | | |
|  | PAAG_01534 | pyruvate dehydrogenase E1 component subunit beta | 289.61 | 5.83 | 0.74 | 1.2.4.1 | Acetyl-CoA biosynthesis | 2 |
|  | PAAG_05761 | 4-carboxymuconolactone decarboxylase family protein | 231.12 | 2.00 | *** | 4.1.1.44 | Aromatic hydrocarbons catabolism | 1 |
|  | PAAG_05572 | conserved hypothetical protein (endo-1,3(4)-beta-glucanase) | 504.06 | 4.67 | *** | 3.2.1.39 | Carbohydrate metabolism | 2 |
|  | PAAG_06473 | mannitol-1-phosphate 5-dehydrogenase | 3302.60 | 16.33 | 0.78 | 1.1.1.17 | Fructose and mannose metabolism | 2 |
|  | PAAG_03776 | inositol-3-phosphate synthase | 1569.78 | 15.33 | 0.79 | 5.5.1.4 | Inostiol biosynthesis | 2 |
|  | PAAG_04181 | sorbitol utilization protein SOU2 | 662.02 | 4.83 | 0.54 | N.A. | Sorbitol metabolism | 2 |
| **Lipid, fatty acid and isoprenoid metabolism** | | | | | | | | |
|  | PAAG_06329 | 3-hydroxybutyryl-CoA dehydrogenase | 427.12 | 8.00 | *** | 1.1.1.157 | Fatty acid metabolism | 2 |
|  | PAAG_05837 | palmitoyl-protein thioesterase | 280.07 | 15.00 | *** | 3.1.2.22 | Fatty acid elongation | 2 |
|  | PAAG_02664 | 3-ketoacyl-CoA thiolase | 2491.03 | 11.17 | 0.80 | 2.3.1.16 | Fatty acid metabolism | 2 |
|  | PAAG_06309 | enoyl-CoA hydratase | 2395.39 | 11.50 | 0.70 | 4.2.1.17 | Fatty acid metabolism | 2 |
|  | PAAG_01928 | peroxisomal dehydratase | 370.76 | 8.75 | 0.37 | N.A. | Fatty acid metabolism | 2 |
| **Metabolism of vitamins, cofactors and prosthetic groups** | | | | | | | | |
|  | PAAG_01324 | folic acid synthesis protein | 351.68 | 10.80 | 0.77 | 4.1.2.25; 2.7.6.3; 2.5.1.15 | Folic acid biosynthesis | 2 |
|  | PAAG_07321 | pyridoxine biosynthesis protein PDX1 | 2963.57 | 12.50 | 0.75 | 4.-.-.- | Pyridoxine biosynthesis | 2 |
| **Secondary metabolism** | | | | | | | | |
|  | PAAG_08820 | conserved hypothetical protein (tyrosinase) | 205.13 | 8.75 | 0.79 | 1.14.18.1 | Melanin biosynthesis | 1 |
|  | PAAG_04888 | 4-coumarate-CoA ligase | 190.87 | 9.00 | *** | 6.2.1.12 | Phenylpropanoids biosynthesis | 1 |
|  | PAAG_00799 | uroporphyrinogen decarboxylase | 362.04 | 8.67 | *** | 4.1.1.37 | Porphyrin biosynthesis | 2 |
|  | PAAG_06925 | conserved hypothetical protein (glutamate-1-semialdehyde 2,1-aminomutase) | 322.93 | 7.00 | *** | 5.4.3.8 | Porphyrin biosynthesis | 2 |
| **ENERGY** | | | | | | | | |
| **Glycolysis and gluconeogenesis** | | | | | | | | |
|  | PAAG_08468 | glyceraldehyde-3-phosphate dehydrogenase | 22116.30 | 24.67 | 0.58 | 1.2.1.12 | Glycolysis/ Gluconeogenesis | 2 |
| **Tricarboxylic-acid cycle** | | | | | | | | |
|  | PAAG_00053 | malate dehydrogenase | 29621.08 | 24.50 | 0.77 | 1.1.1.37 | TCA cycle | 2 |
| **Electron transport and membrane-associated energy conservation** | | | | | | | | |
|  | PAAG_06268 | cytochrome c | 585.65 | 3.80 | 0.70 | N.A. | Electron transport chain | 2 |
|  | PAAG_02699 | hypothetical protein (ubiquinol-cytochrome c reductase subunit 6) | 1636.99 | 2.67 | 0.79 | N.A. | Electron transport chain | 2 |
|  | PAAG_07921 | conserved hypothetical protein (putative cytochrome c oxidase subunit vib protein) | 1384.39 | 2.60 | 0.63 | N.A. | Electron transport chain | 2 |
| **Fermentation** | | | | | | | | |
|  | PAAG_08911 | alcohol dehydrogenase | 315.73 | 9.00 | *** | 1.1.1.1 | Alcoholic fermentation | 2 |
|  | PAAG_04541 | alcohol dehydrogenase | 3967.62 | 12.67 | 0.80 | 1.1.1.1 | Alcoholic fermentation | 2 |
|  | PAAG_00403 | alcohol dehydrogenase | 14799.24 | 21.67 | 0.59 | 1.1.1.1 | Alcoholic fermentation | 2 |
| **Energy conservation and regeneration** | | | | | | | | |
|  | PAAG_05605 | ATP synthase delta chain | 1667.22 | 5.33 | 0.80 | 3.6.3.14 | ATP biosynthesis | 2 |
|  | PAAG_02019 | conserved hypothetical protein (mitochondrial F1F0 ATP synthase subunit Atp14) | 1075.80 | 3.00 | 0.54 | 3.6.3.14 | ATP biosynthesis | 2 |
| **CELL RESCUE, DEFENSE AND VIRULENCE** | | | | | | | | |
| **Stress response** | | | | | | | | |
|  | PAAG_02725 | superoxide dismutase | 508.63 | 5.00 | *** | 1.15.1.1 | Oxidative stress response | 2 |
|  | PAAG_03292 | cytochrome c peroxidase | 371.91 | 7.83 | 0.70 | 1.11.1.5 | Oxidative stress response | 2 |
|  | PAAG_01454 | catalase | 781.72 | 5.20 | 0.37 | 1.11.1.6 | Oxidative stress response | 2 |
|  | PAAG_00293 | quinone oxidoreductase | 232.23 | 4.00 | *** | 1.6.5.5 | Oxidative stress response | 1 |
| **CELL CYCLE AND DNA PROCESSING** | | | | | | | | |
| **DNA processing** | | | | | | | | |
|  | PAAG_08918 | late histone H2B.L4 | 3411.59 | 5.00 | 0.67 | N.A. | Nucleosome assembly | 2 |
|  | PAAG_08917 | histone H2a | 1669.12 | 4.83 | 0.59 | N.A. | Nucleosome assembly | 2 |
|  | PAAG_00427 | conserved hypothetical protein (DNA repair and transcription factor Ada) | 152.88 | 5.00 | *** | N.A. | DNA repair | 1 |
| **Cell cycle** | | | | | | | | |
|  | PAAG_02186 | nuclear segregation protein Bfr1 | 398.41 | 11.00 | 0.73 | N.A. | Mitosis regulation | 2 |
|  | PAAG_03054 | conserved hypothetical protein (G2/M phase checkpoint control protein Sum2) | 202.86 | 7.75 | 0.74 | N.A. | Mitosis regulation | 1 |
|  | PAAG_01298 | M protein repeat protein | 185.34 | 27.00 | *** | N.A. | Mitotic spindle assembly checkpoint | 2 |
| **TRANSCRIPTION** | | | | | | | | |
| **RNA synthesis** | | | | | | | | |
|  | PAAG_08382 | Lamina-associated polypeptide 2 | 389.94 | 7.00 | *** | N.A. | Transcription regulation | 2 |
|  | PAAG_04496 | nascent polypeptide-associated complex subunit beta | 1549.00 | 6.33 | 0.54 | N.A. | Transcription regulation | 2 |
|  | PAAG_01597 | predicted protein (bZIP transcription factor) | 1032.22 | 6.00 | *** | N.A. | Transcription regulation | 2 |
|  | PAAG_02467 | conserved hypothetical protein (transcription initiation factor TFIID subunit 14) | 821.26 | 5.50 | *** | N.A. | Transcription initiation | 2 |
| **RNA processing** | | | | | | | | |
|  | PAAG_04662 | cleavage and polyadenylation specificity factor subunit 5 | 269.70 | 3.00 | *** | N.A. | mRNA processing | 1 |
| **PROTEIN SYNTHESIS** | | | | | | | | |
| **Ribosome biogenesis** | | | | | | | | |
|  | PAAG_05017 | 40S ribosomal protein S10-A | 525.66 | 3.00 | *** | N.A. | Translation | 2 |
|  | PAAG_08634 | 40S ribosomal protein S12 | 449.23 | 6.17 | 0.72 | N.A. | Translation | 2 |
|  | PAAG_05704 | 40S ribosomal protein S13-1 | 1500.97 | 7.40 | 0.79 | N.A. | Translation | 2 |
|  | PAAG_04690 | 40S ribosomal protein S15 | 7159.23 | 3.67 | 0.72 | N.A. | Translation | 2 |
|  | PAAG_05778 | 40S ribosomal protein S19 | 2639.44 | 5.83 | 0.64 | N.A. | Translation | 2 |
|  | PAAG_03322 | 40S ribosomal protein S20 | 831.39 | 3.00 | 0.70 | N.A. | Translation | 2 |
|  | PAAG_05805 | 40S ribosomal protein S21 | 3021.22 | 4.20 | 0.77 | N.A. | Translation | 2 |
|  | PAAG_06882 | 40S ribosomal protein S24 | 854.62 | 5.20 | 0.67 | N.A. | Translation | 2 |
|  | PAAG_01785 | 40S ribosomal protein S3 | 2242.64 | 12.83 | 0.80 | N.A. | Translation | 2 |
|  | PAAG_07707 | 60S ribosomal protein L10a | 318.92 | 9.50 | *** | N.A. | Translation | 1 |
|  | PAAG_04425 | 60S ribosomal protein L22 | 3250.68 | 6.60 | 0.73 | N.A. | Translation | 2 |
|  | PAAG_05233 | 60S ribosomal protein L26 | 1240.01 | 6.00 | *** | N.A. | Translation | 2 |
|  | PAAG_01050 | cytosolic large ribosomal subunit protein L30 | 920.25 | 4.20 | 0.72 | N.A. | Translation | 2 |
|  | PAAG_09096 | hypothetical protein (40S ribosomal protein S28) | 4232.84 | 3.17 | 0.74 | N.A. | Translation | 2 |
| **Translation** | | | | | | | | |
|  | PAAG_02024 | elongation factor 1-alpha | 346.65 | 4.00 | *** | N.A. | Translation elongation | 2 |
|  | PAAG_00241 | eukaryotic translation initiation factor 5A | 289.07 | 3.00 | *** | N.A. | Translation initiation | 2 |
|  | PAAG_00240 | eukaryotic translation initiation factor 5A | 538.18 | 6.67 | 0.78 | N.A. | Translation initiation | 2 |
|  | PAAG_06623 | translation initiation factor 4B | 207.36 | 8.67 | 0.56 | N.A. | Translation initiation | 1 |
|  | PAAG_00772 | hypothetical protein (eukaryotic translation initiation factor 3 subunit J) | 182.70 | 1.00 | *** | N.A. | Translation initiation | 1 |
|  | PAAG_05882 | translation factor SUI1 | 907.08 | 3.50 | *** | N.A. | Translation regulation | 2 |
| **Aminoacyl-tRNA-synthethases** | | | | | | | | |
|  | PAAG_08172 | lysyl-tRNA synthetase | 150.31 | 10.00 | *** | 6.1.1.6 | Aminoacyl-tRNA biosynthesis | 1 |
| **PROTEIN FATE (folding, modification, destination)** | | | | | | | | |
| **Protein folding and stabilization** | | | | | | | | |
|  | PAAG_00739 | peptidyl-prolyl cis-trans isomerase B | 8036.89 | 11.00 | 0.80 | 5.2.1.8 | Protein folding | 2 |
|  | PAAG_07775 | heat shock protein SSB1 | 1838.43 | 14.33 | 0.79 | N.A. | Protein folding | 2 |
|  | PAAG_01778 | peptidyl-prolyl cis-trans isomerase H | 1794.67 | 5.33 | 0.73 | 5.2.1.8 | Protein folding | 2 |
| **Protein targeting, sorting and translocation** | | | | | | | | |
|  | PAAG_07890 | vacuolar-sorting protein snf7 | 206.78 | 4.00 | *** | N.A. | Protein targeting to vacuole | 1 |
| **Protein modification** | | | | | | | | |
|  | PAAG_03038 | ubiquitin-like modifier SUMO | 730.49 | 3.00 | *** | N.A. | Protein sumoylation | 2 |
| **Assembly of protein complexes** | | | | | | | | |
|  | PAAG_01735 | hypothetical protein (cytochrome c oxidase copper chaperone Cox17) | 2250.75 | 2.50 | *** | N.A. | Respiratory chain complex IV assembly | 2 |
|  | PAAG_07543 | conserved hypothetical protein (tubulin-specific chaperone Rbl2) | 678.88 | 2.80 | 0.66 | N.A. | Tubulin complex assembly | 2 |
| **Protein/peptide degradation** | | | | | | | | |
|  | PAAG_04282 | UBX domain-containing protein | 197.43 | 9.00 | *** | N.A. | Proteasomal degradation | 1 |
|  | PAAG_07500 | Xaa-Pro aminopeptidase | 179.14 | 6.00 | *** | 3.4.11.9 | Cytoplasmic and nuclear protein degradation | 2 |
|  | PAAG_04168 | aqualysin-1 | 1117.37 | 7.33 | 0.80 | 3.4.21.111 | Proteolysis | 2 |
|  | PAAG_00664 | aspartyl aminopeptidase | 969.00 | 14.83 | 0.76 | 3.4.11.21 | Proteolysis | 2 |
|  | PAAG_01095 | proline iminopeptidase | 310.95 | 4.50 | 0.75 | 3.4.11.5 | Proteolysis | 1 |
|  | PAAG_05466 | xaa-Pro dipeptidase | 380.68 | 5.75 | 0.74 | 3.4.13.9 | Proteolysis | 2 |
| **PROTEIN WITH BINDING FUNCTION OR COFACTOR REQUIREMENT** | | | | | | | | |
| **Nucleic acid binding** | | | | | | | | |
|  | PAAG_03823 | conserved hypothetical protein (RRM domain-containing protein) | 178.55 | 3.00 | *** | N.A. | RNA binding | 1 |
|  | PAAG_05720 | conserved hypothetical protein (SAP domain-containing protein) | 877.35 | 5.80 | 0.80 | N.A. | Nucleic acid binding | 2 |
| **CELLULAR TRANSPORT, TRANSPORT FACILITIES AND TRANSPORT ROUTES** | | | | | | | | |
| **Transported compounds** | | | | | | | | |
|  | PAAG_00109 | mitochondrial intermembrane space translocase subunit Tim | 854.31 | 4.00 | *** | N.A. | Protein transport | 2 |
|  |  |  |  |  |  |  |  |  |
|  | PAAG_07386 | conserved hypothetical protein (import inner membrane translocase subunit TIM) | 384.76 | 1.00 | *** | N.A. | Protein transport | 2 |
| **Transport routes** | | | | | | | | |
|  | PAAG_06233 | vesicular-fusion protein SEC17 | 161.40 | 5.00 | *** | N.A. | ER to Golgi transport | 1 |
|  | PAAG_08252 | clathrin light chain | 354.63 | 2.20 | 0.70 | N.A. | Vesicular transport | 2 |
|  | PAAG_04651 | GTP-binding nuclear protein GSP1/Ran | 853.14 | 2.17 | 0.39 | N.A. | Nuclear transport | 2 |
|  | PAAG_02465 | hypothetical protein (ran-specific GTPase-activating protein) | 179.71 | 2.00 | *** | N.A. | Nuclear transport | 1 |
| **CELLULAR COMMUNICATION/SIGNAL TRANSDUCTION MECHANISM** | | | | | | | | |
| **Cellular signalling** | | | | | | | | |
|  | PAAG_08247 | calmodulin | 5032.47 | 8.83 | 0.73 | N.A. | Ca2+ mediated signal transduction | 2 |
| **BIOGENESIS OF CELLULAR COMPONENTS** | | | | | | | | |
| **Cell wall** | | | | | | | | |
|  | PAAG_04235 | hydrophobin | 11190.10 | 5.33 | 0.79 | N.A. | Cell wall organization | 2 |
| **CELLULAR COMMUNICATION/ SIGNAL TRANSDUCTION MECHANISM** | | | | | | | | |
| **Transmembrane signal transduction** | | | | | | | | |
|  | PAAG_02817 | stomatin family protein | 192.30 | 5.50 | *** | N.A. | Ion channel mediated signalling pathway | 1 |
| **INTERACTION WITH THE ENVIRONMENT** | | | | | | | | |
| **Homeostasis** | | | | | | | | |
|  | PAAG_02622 | iron-binding protein iscA | 209.72 | 6.00 | *** | N.A. | Iron-sulfur cluster assembly | 1 |
|  | PAAG_02974 | glutaredoxin domain-containing protein | 181.12 | 3.00 | *** | N.A. | Cell redox homeostasis | 1 |
| **SUBCELLULAR LOCALIZATION** | | | | | | | | |
| **Nucleus** | | | | | | | | |
|  | PAAG_08118 | conserved hypothetical protein | 212.37 | 6.00 | *** | N.A. | Lin1 family protein | 2 |
| **UNCLASSIFIED PROTEINS** | | | | | | | | |
|  | PAAG_02217 | isochorismatase domain-containing protein | 1462.05 | 5.20 | 0.69 | N.A. | N.A. | 2 |
|  | PAAG_09108 | RPEL repeat protein | 396.89 | 5.00 | *** | N.A. | N.A. | 2 |
|  | PAAG_09001 | conserved hypothetical protein | 247.80 | 1.00 | *** | N.A. | N.A. | 2 |
|  | PAAG_08409 | hypothetical protein | 2939.15 | 3.00 | *** | N.A. | N.A. | 2 |
|  | PAAG_05403 | predicted protein | 255.76 | 6.00 | *** | N.A. | N.A. | 1 |
|  | PAAG_04178 | conserved hypothetical protein | 178.05 | 2.00 | *** | N.A. | N.A. | 1 |
|  | PAAG_02985 | hypothetical protein | 214.03 | 5.00 | *** | N.A. | N.A. | 1 |
|  | PAAG_02839 | hypothetical protein (HMG box protein) | 342.55 | 8.00 | *** | N.A. | N.A. | 2 |
|  | PAAG_02001 | conserved hypothetical protein | 880.54 | 4.00 | *** | N.A. | N.A. | 2 |
|  | PAAG_01567 | conserved hypothetical protein | 690.12 | 5.00 | *** | N.A. | N.A. | 2 |
|  | PAAG_00467 | predicted protein | 238.33 | 4.00 | *** | N.A. | N.A. | 1 |
|  | PAAG_00026 | conserved hypothetical protein | 328.98 | 6.00 | *** | N.A. | N.A. | 1 |
|  | PAAG_00233 | conserved hypothetical protein (rhodanese domain-containing protein) | 462.00 | 1.00 | *** | N.A. | N.A. | 2 |
|  | PAAG_08015 | hypothetical protein | 1113.98 | 1.00 | 0.80 | N.A. | N.A. | 2 |
|  | PAAG_07772 | conserved hypothetical protein | 1321.21 | 7.50 | 0.74 | N.A. | N.A. | 2 |
|  | PAAG_04083 | conserved hypothetical protein (isochorismatase family hydrolase) | 1368.58 | 3.67 | 0.69 | N.A. | N.A. | 2 |
|  | PAAG_00297 | conserved hypothetical protein (F1F0-ATP synthase regulatory factor Stf2) | 561.22 | 4.25 | 0.59 | N.A. | N.A. | 2 |

^a^Information obtained from *Paracoccidioides* Database (<http://www.broadinstitute.org/annotation/genome/paracoccidioides_brasiliensis/MultiHome.html>)

^b^filter 1 – proteins derived from PepFrag2; filter 2 – proteins derived from PepFrag1, as determined by PLGS and cited by Murad and Rech (2012).

***: proteins identified just in presence of inorganic iron;

N.A.: not applicable.
